# Differentiation-induced reduction in functional diversity restricts the ability of cytomegalovirus-specific CD8 T cells to eliminate virus-infected cells

**DOI:** 10.1016/j.ebiom.2025.106107

**Published:** 2026-01-05

**Authors:** Lea Fritz, Ahmed Hassan, Lennart Riemann, Berislav Čuvalo, Bibiana Costa, Britta Wieland, Britta Eiz-Vesper, Christine Falk, Lennart M. Roesner, Thomas Werfel, Ulrich Kalinke, Hristo Georgiev, Reinhold Förster, Berislav Bošnjak

**Affiliations:** a Institute of Immunology, Hannover Medical School, Hannover, Germany; b Department for Paediatric Pneumology, Allergology and Neonatology, Hannover Medical School, Hannover, Germany; c Institute for Experimental Infection Research, TWINCORE, Centre for Experimental and Clinical Infection Research, A Joint Venture Between the Helmholtz Centre for Infection Research and the Hannover Medical School, Hannover, Germany; d University Women's Hospital, Hannover Medical School, Hannover, Germany; e Institute of Transfusion Medicine and Transplant Engineering, Hannover Medical School, Hannover, Germany; f German Centre for Infection Research (DZIF), Partner Site Hannover-Braunschweig, Hannover, Germany; g Institute of Transplantation Immunology, Hannover Medical School, Hannover, Germany; h German Centre for Lung Research (DZL), BREATH Site, Hannover, Germany; i Department of Dermatology and Allergy, Hannover Medical School (MHH), Hannover, Germany; j Cluster of Excellence RESIST (EXC 2155), Hannover Medical School, Hannover, Germany

**Keywords:** CD8 T cells, Differentiation, Functional diversity, Human cytomegalovirus, Infected cell killing, pp65

## Abstract

**Background:**

Human cytomegalovirus (HCMV) is one of the pathogens with the most significant impact on the immune system's composition, including the expansion of virus-specific CD8 T cells. Nevertheless, it remains unclear why individuals with expanded CD8 T cells recognising the pp65-HLA-A^∗^02:01–restricted viral epitope NLVPMVATV (NLV-T cells) exhibit weakened immune control of HCMV reactivation.

**Methods:**

Here, we characterised NLV-T cells from 116 healthy HCMV-positive donors, dividing them into two groups: those with low and those with high NLV-T cell frequencies (LF and HF, respectively). We phenotyped the cells using multi-colour spectral flow cytometry and single-cell RNA sequencing coupled with TCR profiling and examined their killing properties against peptide-loaded and virus-infected target cells.

**Findings:**

Our comprehensive multimodal analysis revealed that NLV-T cells from HF donors exhibited a phenotype of advanced differentiation, marked by high levels of granzyme B and perforin expression, and efficiently eliminated peptide-loaded targets and HCMV-infected cells as long as cell surface HLA expression was unaffected. However, NLV-T cells from LF donors, possessing a less differentiated granzyme K-intermediate phenotype, demonstrated enhanced cytokine secretion and the ability to eliminate HCMV-infected cells, even in the presence of virus-induced HLA class-I downregulation.

**Interpretation:**

Overall, these findings suggest that HCMV exploits CD8 T cell differentiation to evade immune protection. These data are crucial for understanding the previously observed decline in HCMV reactivation control in individuals with NLV-T cell accumulation. Moreover, our findings have clinical implications and could guide future research on adoptive T-cell therapy, and the potential use of HCMV as a vaccine vector.

**Funding:**

10.13039/501100001659Deutsche Forschungsgemeinschaft (DFG, German Research Foundation)–Projects number 390874280 and FO334/7-2.

**Research in context**

**Evidence before this study**

HCMV establishes persistent and then latent infection that lasts throughout the host's lifetime. During latent HCMV infection, presence of viral antigen leads to expansion of HCMV-specific CD8 T cells. However, individuals with expanded CD8 T cells may experience weakened immune control of HCMV reactivation, which correlates to potential complications such as increased risk of coronary disease and frailty.

**Added value of this study**

To provide deeper insight in the mechanisms why expanded HCMV-specific T cells lose their ability to protect against HMCV, we phenotypically and functionally compared HCMV-specific T cells between donors with their low and high frequency in blood. We found that HCMV-specific T cell from donors with their high frequency in blood express high levels of granzyme B, perforin, and KLRG1 and exhibit potent cytotoxic activity against peptide-loaded targets and HCMV-infected cells, but only when HLA expression was intact. In contrast, they were impaired in their ability to kill HCMV-infected cells that had evaded immune detection through HLA downregulation. Conversely, NLV-T cells from LF donors secreted higher levels of cytokines IFNγ and TNF, and were more effective at eliminating HCMV-infected cells that had suppressed HLA expression, highlighting a potential advantage of the this cell population in responding to HCMV infection.

**Implications of all the available evidence**

Our study elucidates the complex interplay between CD8 T cells and HCMV and describes a previously unrecognised mechanism by which HCMV exploits the differentiation of CD8 T cells to reduce functional CD8 T cell diversity and thus evade immune protection. These data provide a deeper understanding of the factors that contribute to diminished control of HCMV reactivation in individuals with a high frequency of HCMV-specific CD8 T cells that have an increased risk of coronary diseases, frailty, and mortality.

## Introduction

In response to most viral infections, virus-specific CD8 T cells initially expand and, after pathogen eradication, contract and establish immunological memory. However, the HCMV or human herpesvirus type 5 (HHV-5) evolved immune escape mechanisms, allowing the virus to establish persistent and then latent infection throughout the host's lifetime.^1^ Continued exposure to viral antigens induces long-term maintenance or even slow expansion of HCMV-specific CD8 T cells, a phenomenon termed “memory inflation”.^2^ As a result, HCMV-specific CD8 T cells constitute a significant proportion of circulating CD8 T cells, averaging 5–10% in healthy individuals, particularly in the elderly population.^3^^,^^4^ The expanded HCMV-specific T cells have a distinct advanced differentiation state.^5^^,^^6^ They express a combination of adhesion, homing, and effector molecules that phenotypically render these cells as a mixture of effector memory T cell (Tem) and effector memory T cells re-expressing CD45RA (TEMRA).^2^^,^^5^ These cells are not exhausted and express high levels of granzyme B, perforin, and the cytokines interferon-gamma (IFNγ) and tumour necrosis factor (TNF) after re-stimulation.^7^^,^^8^

Despite increased numbers of highly functional CMV-specific T cells in circulation, evidence indicates that immune control of HCMV replication after reactivation is diminished in older individuals. Viral DNA, which is usually undetectable in HCMV-seropositive healthy individuals, has been found in the urine, blood, or blood cells of elderly individuals.^9^, ^10^, ^11^, ^12^ While there have been no reported cases of clinically significant HCMV reactivation in healthy elderly individuals, we and others have shown that the increased number of HCMV-specific CD8 T cells in circulation is associated with an increased risk of coronary diseases, frailty, and mortality.^13^, ^14^, ^15^, ^16^ In contrast, HCMV seropositivity in elderly individuals does not appear to be linked to any adverse health outcomes.^17^ Collectively, these findings suggest that the failure of expanded HCMV-specific CD8 T cells to control viral replication could be a contributing factor to reduced life expectancy in HCMV-seropositive elderly individuals.

Two of the most immunodominant antigens to which HCMV-specific CD8 T cells respond are the 72-kDa immediate early 1 protein (IE-1; UL123) and the tegument protein pp65 (UL83).^18^ Recent study has demonstrated that the expansion of IE-1-specific CD8 T cells is accompanied by a reduction in the population's TCR structural affinity, as a consequence of the proliferation of cells with low-affinity T cell receptors (TCRs).^19^ The TCR sequence plays a crucial role in shaping T cell fate and function.^20^ Consequently, memory inflation of IE-1-specific T cells has been proposed as a compensatory mechanism–referred to as reverse TCR repertoire evolution–to preserve the overall population functionality despite the low intrinsic TCR structural affinity.^19^ However, these findings do not explain the lack of HCMV control in individuals where IE-1-specific CD8 T cells are absent or constitute only a small fraction of the virus-specific CD8 T cell population.^18^^,^^21^, ^22^, ^23^ Furthermore, CD8 T cells specific to pp65, which exhibit similar frequencies and phenotypes to those targeting IE-1,^18^^,^^21^, ^22^, ^23^ have been shown to possess TCRs with high antigen affinity.^24^, ^25^, ^26^ In contrast to IE-1-specific CD8 T cell responses, expansion of pp65-specific CD8 T cells is believed to be a consequence of infrequent subclinical occurrences of HCMV reactivation.^23^ Moreover, pp65-specific CD8 T cells maintain their proliferation capacity and cytokine production in elderly individuals.^8^^,^^27^ Therefore, the concept of reverse TCR repertoire evolution does not seem to explain the age-related accumulation of a large numbers of CD8 T cells recognising the pp65-HLA-A^∗^02:01–restricted viral epitope NLVPMVATV (NLV-T cells).^28^^,^^29^ Furthermore, there are conflicting reports regarding the functionality of the expanded NLV-T cell populations. While some reports described that expanded NLV-T cells are multifunctional,^3^^,^^30^ others described impaired effector functions in these cells.^29^^,^^31^^,^^32^

We hypothesised that the expansion of NLV-T cells might be a result of their diminished functionality. Therefore, we conducted an in-depth characterisation of NLV-T cells from donors with low and high NLV-T cell frequency in the blood. Our analysis revealed that NLV-T cell expansion affects their differentiation, clonality, and the production of effector molecules. NLV-T cells from HF donors, characterised by the expression of granzyme B, perforin, and KLRG1, efficiently eliminated peptide-loaded targets and HCMV-infected cells as long as cell surface HLA class-I expression was unaffected. However, they were unable to kill virus-infected cells in which HCMV-encoded immune evasion mechanisms resulted in down-regulation of surface HLA class-I molecules. In contrast, NLV-T cells from LF donors secreted higher levels of IFNγ and TNF and were more potent in eliminating HCMV-infected cells with virus-induced HLA class-I downregulation. Together, these findings suggest that the expansion of too specifically differentiated NLV-T cells could be a compensation mechanism trying to overcome their functional impairment in eliminating HCMV-infected cells. Hence, our data offer new insights into the protective mechanisms of HCMV-specific CD8 T cells and support the selection of virus-specific T cells with less differentiated phenotypes for adoptive transfer therapy in immunosuppressed patients with HCMV infection.

## Methods

### Study participants and approval

We used frozen peripheral blood mononuclear cell (PBMC) samples from the RESIST senior individual (SI) cohort. This cohort comprises 550 individuals aged 60 years or older and 100 individuals aged 20–40 years with a permanent residence in Hannover who were enrolled in the study between December 2019 and March 2022. Exact cohort description, including the inclusion and exclusion criteria, was described in detail earlier.^33^ For this study, we randomly selected 110 samples from SI that were CMV-seropositive and expressed HLA-A^∗^02 on blood cells, as determined previously using flow cytometry.^16^ Of those, 23 randomly selected samples were fully HLA-typed. Additionally, we included 21 samples from CMV-seropositive and HLA-A^∗^02-positive platelet donors from the Institute of Transfusion Medicine and Transplant Engineering at Hannover Medical School who provided written informed consent. We included these samples from both LF and HF donors for two primary reasons: (i) to augment the number of donors under the age of 60, as the RESIST SI cohort is specifically focused on elderly individuals, and (ii) to obtain samples with high cell numbers that would enable their subsequent analysis in multiple functional tests. From all platelet donors, we had complete HLA haplotypes, as they are routinely HLA-characterised.

### Ethics

The use of blood samples was approved by the local Institutional Review Board (Ethikkommission der Medizinischen Hochschule Hannover; 3639-2017 and 8615_BO_S_2019) and written consent was obtained from all enrolled participants.

### Sex as a biological variable

We included samples from male and female donors in our study and our results were independent of donor sex assigned at birth (Supplementary Fig. S3A).

### Blood sample acquisition and preparation

The department of transfusion medicine of the Hannover Medical School provided platelet aphaeresis disposable kits remaining after routine platelet collection. Blood was extracted from the kits and PBMCs were isolated using density centrifugation (Pancoll human; PAN Biotech). Isolated cells were counted and resuspended in a freezing medium [90% foetal bovine serum (FBS; Sigma–Aldrich) and 10% DMSO (Sigma–Aldrich)], and frozen at −80 °C until analysed further.

### Cell lines

THP-1 cells (ATCC TIB-202; RRID: CVCL_0006) were cultured in RPMI-1640 medium (Gibco) supplemented with 10% heat-inactivated FBS, 100 U/mL Penicillin-Streptomycin, 2 mM l-Glutamine (Gibco), and 0.05 mM β-mercaptoethanol. MRC-5 cells (ATCC CCL-171; RRID: CVCL_0440) were cultured in 80% DMEM (Capricorn) supplemented with 10% heat-inactivated FBS, 2 mM GlutaMax, 1% sodium pyruvate (Gibco), and 20% FibroGro Medium (Merck). All cells were kept in a Heraeus BBD 6220 incubator (Thermo Scientific) at 37 °C, with 5% CO_2_ and 95% relative humidity.

### HCMV virus stocks

In these experiments, two different HCMV reporter viruses were used. The HCMV variant TB40-BAC_KL7_-SE-EGFP-UL40rep (HCMV-WT)^34^ was generated on the basis of the TB40-BAC_KL7_-SE-EGFP clone.^35^ Briefly, using the method of Tischer et al., a vector cassette was inserted into the backbone to replace the US2, US3, and US6 genes.^36^ Next, the UL40 open reading frame (ORF) was repaired and a self-excising (SE) vector cassette was relocated into the middle of the UL74 ORF via markerless mutagenesis. The insertion of the EGFP expression cassette downstream of US34A was done via markerless recombination. The HCMV mutant TB40-BACKL7-SE-EGFP-(ΔUS2-6+ΔUS11)-UL40rep lacking the genes encoding for immunoevasins US2-6 and US11 (HCMV-ΔUS2-6+11) was generated by omitting the replacement of the US2-6 region of the TB40-BACKL7 clone and by additional deletion of the US11 region.^37^

Viral titres were determined on MRC-5 cells by an indirect immunoperoxidase labelling procedure.^38^ In brief, MRC-5 cells were infected with 10-fold serial dilutions of HCMV, centrifuged at 300×*g* for 30 min and incubated for 3 days. MRC-5 were fixed with methanol for a minimum of 1 h at −20 °C. The cells were incubated for 30 min with the primary antibody directed against cytomegalovirus immediate early and early nuclear proteins (#M0854, 1:100, Dako) and then for another 30 min with the secondary goat anti-mouse-HRP antibody (#5450-0011, 1:500, KPL). Afterwards the cells were incubated with the substrate AEC (#925804 and #925903, 1:50, Biolegend) for 20 min. Infected cells were microscopically counted and viral titres were calculated. These titres were used to calculate the multiplicity of infection (MOI). For infection, the cells were either exposed to HLA-hi, HLA-low or left untreated at MOI of 3. To infect the THP-1 macrophages, the virus was added to the culture medium and the plates were centrifuged at 300 g for 30 min (at RT).

### Expansion of NLV-T cells from PBMCs

PBMCs and NLV-T cells were cultured in Advanced RPMI medium (Gibco) supplemented with 10% heat-inactivated FBS, 100 U/mL Penicillin-Streptomycin (Gibco), 2 mM GlutaMax (Gibco), and 0.05 mM β-mercaptoethanol (Sigma). The medium was freshly supplemented with 100 IU/mL IL-2 (Sigma–Aldrich) before use.

For NLV-T cell expansion, PBMCs were thawed in a 37 °C water bath and washed with PBS twice. After washing, the cells were rested in a culture medium without IL-2 for 4 h at a concentration of 2 x 10^6^ cells/mL at 37 °C. After the resting phase, the cells were resuspended at a concentration of 1 × 10^6^ cells/mL in a medium supplemented with IL-2 and stimulated with the HCMV pp65 (495–503) HLA-A^∗^02:01 NLVPMVATV (NLV) peptide (Gibco) at a final concentration of 0.1 μM. The cells were seeded into 6-well plates and incubated for expansion. On day 3 of culture, half of the culturing medium was replaced with fresh IL-2 medium. On day 5 of culture, cells were split 1:1 into fresh 6-well plates. On day seven of the culture, the cells were counted and transferred into cell culture flasks at a concentration of 1 × 10^6^ cells/mL. From day seven on, the cells were counted and the density was adjusted back to 1 × 10^6^ cells/mL every other day.

If not stated otherwise, to obtain pure NLV-T cells for experiments, we isolated them at day 9 of culture using NLV-peptide-loaded MHC I-Streptamers (MHC I-Strep HLA-A^∗^0201; CMV pp65, NLVPMVATV; Iba), conjugated to Strep-Tactin Magnetic Microbeads (Strep-Tactin® Magnetic Microbeads, Iba), according to the protocol described in the user manual.

### Spectral flow cytometry analysis of NLV-T cell phenotype

To detect NLV-T cells, PBMCs (immediately after thawing) were labelled with conventional HLA-A^∗^0201 tetramers (BV650-labelled; Tetramer Shop, Cat.No. HA02-070) loaded with NLVPMVATV peptide (GenScript) and additionally with NLV-peptide-loaded HLA-A^∗^0201 “null-tetramers” (PE-labelled; MBL). Initially, we labelled a few samples of *ex vivo* proliferated cells with the same tetramer combination. Afterwards, cells from the culture were labelled with conventional NLV-peptide loaded PE-coupled tetramers (NIH Tetramer Core Facility of Emory University (Atlanta, GA)).

Tetramer-labelling was performed for 15 min at 37 °C, followed by extracellular labelling for 25 min at RT with Panel 1 antibodies (Supplementary Table S2). After washing, the samples were acquired on a Cytek Aurora spectral flow cytometer (Cytek) equipped with five lasers (355 nm, 405 nm, 488 nm, 561 nm, and 640 nm). Spectral unmixing was done with SpectroFlo (Cytek). NLV-T cell phenotypes were analysed using an unsupervised clustering analysis approach as described earlier.^39^^,^^40^ Briefly, flow cytometry data was first pre-gated on CD14^−^CD19^−^CD3^+^CD8^+^Tet-NLV^+^ live singlet cells using FlowJo software v. 10.7.1 (BD Bioscience), exported from FlowJo as.fcs files, and then loaded into R (version 4.3.2). Arcsin transformation was performed using the flowVS package.^41^ The FlowSOM algorithm was run on the transformed dataset to cluster cells based on their surface marker expressions in an unsupervised manner.^42^ Clusters were then manually assessed and annotated based on their respective marker expressions. The Uniform Manifold Articles Approximation and Projection (UMAP) approach was used for dimensionality reduction.

### Preparation of NLV-T cell single-cell RNA sequencing (scRNAseq) datasets

PBMCs from 8 LF were thawed, and CD8 T cells were enriched by negative selection (CD8+ T cell isolation kit, Miltenyi Biotec). PBMCs from 12 HF donors were processed immediately after thawing. The cells from both donor groups were labelled with conventional NLV-peptide loaded PE-coupled tetramers (NIH Tetramer Core Facility of Emory University, Atlanta, GA), for 15 min at 37 °C. Then, cells were labelled for 25 min at room temperature with fluorescently-labelled antibodies, Totalseq antibodies, and cell-hashing antibodies listed as Panel 2 in Supplementary Table S2. After washing, up to 8 samples from LF or HF donors labelled with distinct cell-hashing antibodies were pooled, and cells were sorted for CD19- CD14- CD16- CD56- CD3+ CD8+ NLV-tetramer + cells on a FACSAria III Fusion or FACSAria IIu cell sorter (both BD). A total of three NLV-T cell pools were loaded onto the Chromium iX (10× Genomics), and used to generate three independent sequencing libraries using 10× Genomics Chromium Next GEM Single Cell 5′ Reagent Kits v2 according to the manufacturer's protocol (user guide CG000330 Rev F). Generated sequencing libraries were sequenced on NovaSeq X (Illumina) using PE150 configuration.

### scRNAseq data analysis

Generated FASTQ files were processed using Cell Ranger (v7.0.1; 10x Genomics) multi pipeline using the default parameters (intronic reads included). Seurat (v4.3.0.1) was used to import the Gene/Hashtag count matrices. VDJ information was later incorporated to Seurat objects as metadata using djvdj (v0.1.0; https://github.com/rnabioco/djvdj/). To clean the dataset we removed the ambient RNA counts using SoupX (v1.6.2) as well as cells with high mitochondrial gene percentage.^43^ Hashtag demultiplexing and doublet removal were done using MULTIseqDemux function from Seurat and scDblFinder (v1.18.0; https://github.com/plger/scDblFinder), respectively. Latent representations computed by scVI (v1.0.0; https://github.com/YosefLab/scVI) based on the top 2000 variable genes were used to build the integrated UMAP. Differentially gene expression between clusters was performed using the Findallmarkers function of Seurat with MAST (v1.26.0; https://github.com/RGLab/MAST) method. TCR affinities were predicted using pMTnet package (https://github.com/tianshilu/pMTnet). A seed was set to “44” for R and “20” for scVI for reproducibility. Data visualization was done using SCpubr (v1.1.2; https://enblacar.r-universe.dev/SCpubr).

### Bulk RNA and TCR sequencing

RNA was isolated from the expanded and MHC-I-streptamer-sorted NLV-T cells using RNeasy Plus Mini Kit (Qiagen) according to manufacturer's protocol. RNA quality and concentration were assessed using a 2100 Bioanalyzer (Agilent). Bulk RNA sequencing libraries were generated from 100 ng RNA of each sample using NEBNext Ultra II RNA Library Prep Kit (NEB) according to the manufacturer's protocol. After quality control using 2100 Bioanalyzer (Agilent), the generated sequencing libraries were sequenced on NovaSeq X (Illumina) using PE150 configuration.

To generate TCR sequencing libraries, 100 ng of isolated RNA per sample served as a starting material to generate cDNA libraries using SMART-Seq Human TCR (with UMIs) (Takara Bio) according to the manufacturer's protocol. Quality-controlled TCR sequencing libraries (2100 Bioanalyzer; Agilent) were sequenced on MiSeq (Illumina) using PE300 configuration.

For analysis of bulk RNA libraries, generated FASTQ files were pseudo-aligned to the human genome reference (ENSEMBL GRCh38, version 111) using Kallisto (v 0.50.1; https://github.com/pachterlab/kallisto) to quantify transcripts. Kallisto outputs were imported to R (v4.3.2) using tximport (v1.32.0; https://github.com/thelovelab/tximport) and filterByExpr function from edgeR (v3.32.1; https://bioconductor.org/packages/release/bioc/html/edgeR.html) was used to filter outliers. DESeq2 (v1.44.0; https://bioconductor.org/packages/release/bioc/html/DESeq2.html) was used to identify differentially expressed genes between HF and LF samples. Data visualization was done using EnhancedVolcano (v1.22.0; https://bioconductor.org/packages/release/bioc/html/EnhancedVolcano.html).

Generated FASTQ files from TCR sequencing libraries were annotated using MIXCR (v4.6.0; https://github.com/milaboratory/mixcr) and further data analysis and visualization was done using immunarch (v1.0.0; https://zenodo.org/records/10840553). TCR affinity scores of the top two expanded NLV-T cell clones from each donor were calculated using the pMTnet package (https://github.com/tianshilu/pMTnet). CDR3 sequence alignments were performed using MUSCLE (multiple sequence comparison by log-expectation; http://www.drive5. com/muscle)^44^ and illustrations of consensus sequences were created with WebLogo (http://weblogo.berkeley.edu/logo.cgi).^45^

### Effector molecule concentration determination

We used the LEGENDplex™ Multi-Analyte Flow assay Kit (Biolegend) to determine concentrations of the following targets: IL-2, IL-4, IL-10, IL-6, IL-17A, TNF, sFas, sFasL, IFNγ, granzyme A, granzyme B, perforin, and granulysin. Briefly, the frozen supernatants from indicated co-cultures of NLV-T cells with divergent target cells were thawed, diluted 1:5 with assay buffer, and incubated with capture beads for 2 h, followed by incubation with the detection antibodies for 1 h. Samples from killing assays with HCMV-infected targets were fixed with 1% paraformaldehyde (PFA) in water for 10 min before acquisition. The samples were acquired on a spectral flow cytometer according to the manufacturer's instructions. Finally, analyte concentrations were calculated using LEGENDplex™ data analysis software (Biolegend).

### Restimulation of *ex vivo* expanded NLV-T cells to assess effector molecule production

We used intracellular labelling for perforin, granzyme B, IFNγ, and TNF to assess the effector molecule profile of NLV-T cells. At day 7 of NLV-T cell expansion from PBMCs, we mixed expanded cells as in E:T of 1:4 with non-loaded THP-1 cells or THP-1 cells loaded with 10 μM NLV-peptide. The cells were co-cultured on U-shaped 96-well plates in the presence of Brefeldin A (Sigma) at a final concentration of 5 μg/mL. For the positive control, T cells were stimulated with phorbol 12-myristate 13-acetate (PMA; 5 μg/mL; Calbiochem) and Ionomycin (1.5 μg/mL; Invitrogen), also in the presence of Brefeldin A. After overnight incubation, the cells were collected, labelled with NLV-tetramers, surface markers, and intracellular markers (Panel 3 in Supplementary Table S2).

For intracellular labelling, we used the Invitrogen intracellular Fix & Perm Set (Thermo Fisher Scientific) according to the manufacturer's instructions. Briefly, after tetramer labelling and labelling of extracellular markers, the cells were fixed with the fixation buffer for 30 min at room temperature. Following fixation, the cells were washed with permeabilization buffer, and labelled with the antibodies for effector molecules for 45 min at room temperature. Afterwards, the cells were washed with MACS buffer (PBS + 3% FCS + 2 mM ethylene diamine tetra-acetic acid (EDTA)) and acquired utilising spectral flow cytometry.

### NLV-T cell degranulation

The expanded NLV-T cells were restimulated with either plate-bound anti-CD3 and anti-CD28 antibodies (4 μg/mL and 1 μg/mL, respectively) or NLV-peptide-loaded MRC-5 cells. MRC-5 cells were seeded to 96-well flat-bottom plates, incubated overnight to attach, and then loaded with the NLV-peptide for 2 h before NLV-T cells were added in a 1:1 E:T ratio. The NLV-T cells were then incubated for 1 and 2 h with either the anti-CD3/anti-CD28 antibodies or the peptide-loaded targets. The cells were collected and labelled with Panel 4 (Supplementary Table S2). As a control, we used cells that were not stimulated. Cells were acquired on the Cytek Aurora spectral flow cytometer (Cytek). The median fluorescence intensity (MFI) of the CD107a signal on CD3^+^CD8^+^cTet^+^ NLV-T cells was determined in FlowJo. To calculate fold change in CD107a MFI, we set the CD107a MFI value of control (non-stimulated) cells from each donor at 1.

### Tetramer avidity assay

The expanded NLV-T cells were labelled with a 2-fold dilution series of nTet. The final nTet amounts per labelling ranged from 5 pmol to 0.78 pmol. Labelling was conducted in PBS for 15 min at 37 °C, followed by labelling with anti-CD3 and anti-CD8 antibodies for 25 min at room temperature (listed in Panel 1 in Supplementary Table S2). After washing, the cells were acquired with the Cytek Aurora spectral flow cytometer (Cytek), and data was analysed using the FlowJo software. The MFI of the nTet labelling in the respective dilutions was compared between samples. The half-maximal nTet binding amount was calculated using the LD50 calculator from AAT Bioquest (https://www.aatbio.com/tools/ld50-calculator).

### Functional avidity assay

For functional avidity testing, the xCELLigence RTCA SP system together with the RTCA E-plates (Agilent) or the Maestro Z (Axion Biosystems) was used. Both systems measure electrical resistance (impedance) to monitor cell density in real-time and follow similar protocols. Initially, the machines were calibrated with a medium-filled plate to obtain background measurements. On day one of the experiment, MRC-5 cells, that were used as target cells, were plated (24,000 cells/well in 200 μL) and incubated overnight to allow attachment to the plate. After 24 h, NLV-peptide was added to the cells in the concentration range from 1 μM to 10^−5^ μM in 10-fold dilution steps. After 2 h of peptide loading, 6000 *ex vivo* expanded, steptamer-isolated NLV-T cells were added to each well (E:T ratio 1:4). NLV-T cells were previously incubated with 8.75 μg of a CD8-blocking antibody (Biolegend; Supplementary Table S2) for 15 min.

After NLV-T cells were added, the impedance was measured for at least 18 h. The obtained data was analysed using the RTCA Software Pro (Aglient) or AxIS Z-GxP-3.10.3 (Axion Biosystems). For the quantification of NLV-T cell killing, all impedance values were normalised to the last time-point before T cell addition and were set as 0. Next, we calculated the mean area under the curve (AUC) values of each test group (NLV-loaded MRC-5 cells co-cultured with NLV-T cells) on impedance (measured as cell index) *vs.* time graphs (encompassing 18 h after the addition of NLV-T cells). The percentage of killed target cells exposed to the NLV-T cells from each donor was determined by dividing the mean AUC value of the wells exposed to NLV-T cells with the AUC value from untreated MRC-5 cells.

### NLV-T cell killing of HCMV-infected macrophages

THP-1 cells were stimulated using a medium containing 200 nM PMA to induce differentiation into macrophages. After two days, the culturing medium was replaced with fresh RPMI. On day three, the differentiated macrophages were harvested by incubation with 2 mM EDTA in PBS for 15 min on ice. Next, 50,000 macrophages were seeded per well and infected using spinoculation with either the HCMV-WT or the HCMV-ΔUS2-6+11. We used the multiplicity of infection of 3 for both viruses. The cells were maintained in the culture for three days before the NLV-T cells were added in ratios of 1:1, 1:4, and 1:16. After overnight co-culture, the cells were harvested, fixed, and analysed with spectral flow cytometry. Supernatants were harvested and frozen until effector molecule concertation was determined.

### Statistical analysis

We performed statistical analyses using GraphPad Prism (version 8) and indicated used statistical tests in the Figure legends. Briefly, to compare phenotype of NLV-T cells from LF and HF donors we used unpaired t-test with Welch's correction. To compare NLV-T cell percentage between male and female donors, we used Welch's ANOVA followed by Dunnett's T3 multiple comparison test. Distribution of NLV-T cells within the scRNAseq clusters was analysed using two-way RM ANOVA followed by a two-stage linear step-up procedure of Benjamini, Krieger, and Yekutieli. For other comparisons of single parameter between NLV-T cells between LF and HF donors, we used unpaired t-test with Welch's correction. To analyse distribution of NLV-T cell subpopulations between LF and HF donors, we used two-way ANOVA followed by Sidak's multiple comparison test and indicated factors in the figure legends. We used simple linear regression to correlate NLV-T cell frequencies with other parameters. We considered p values ≤ 0.05 statistically significant.

### Role of founders

The funders had no involvement in the study design, data collection, data analysis, data interpretation, or manuscript preparation.

## Results

### NLV-T cells from HF donors have a mixture of Tem and TEMRA phenotypes

**Fig. 1:**
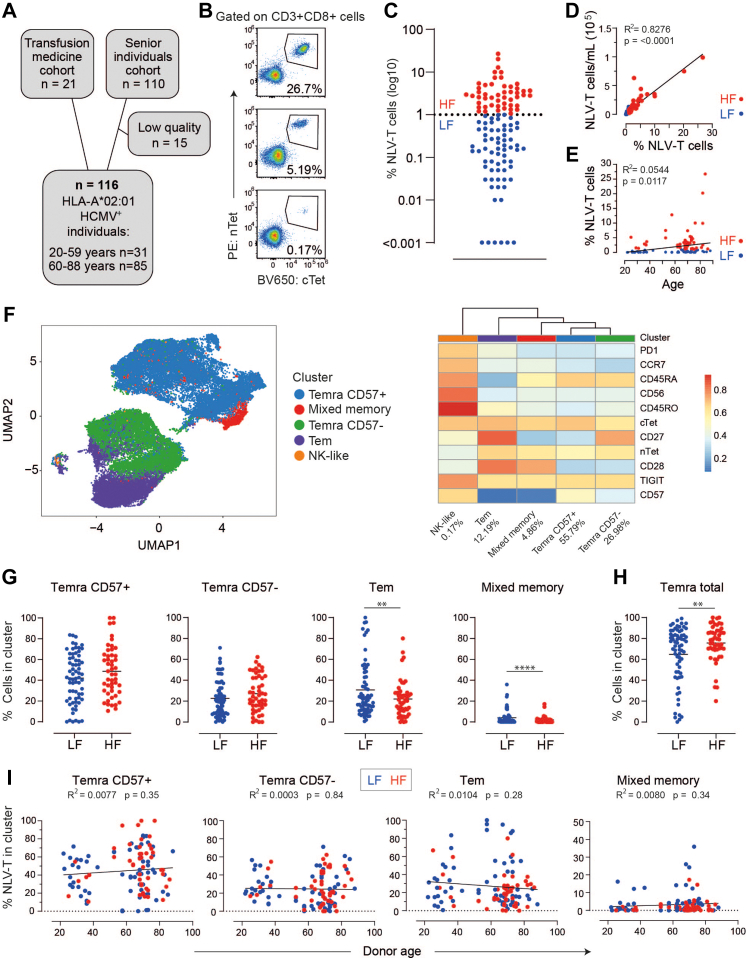
**HCMV-induced accumulation increases the number of NLV-T cells with TEMRA phenotype**. **A**) Patient cohort overview. **B**) Spectral flow cytometry data showing frequency of T cells labelled with conventional (cTet) and null-tetramers (nTet) NLV (NLV-T cells) within CD3^+^CD8^+^ cells. Cells were labelled with Panel 1a antibodies from Supplementary Table S2 and gated as depicted in Supplementary Fig. S1A. Representative examples from three different donors. **C**) According to the NLV-T cell frequency within the CD3 + CD8+ T cell gate, 116 donors were split into high-frequency (NLV-T cells >1%, HF, n = 49) and low-frequency (NLV-T cells <1%, LF, n = 67) groups. **D, E**) NLV-T cell frequencies within the CD3^+^CD8^+^ T cell gate correlated with (**D**) number of NLV-T cells/mL of blood and (**E**) weakly with donor. Dots–data from individual donors, line–simple linear regression. **F**) NLV-T cell composition presented as UMAP (left) and heatmap of individual marker expression (right) generated from concatenated NLV-T cells from all donors. **G**) Frequencies of NLV-T cells from LF and HF donors in the individual clusters. **H**) Frequency of NLV-T cells within both (CD57^+^ and CD57^−^) TEMRA clusters. **G, H**) Unpaired t-test with Welch's correction: ∗∗ p < 0.01; ∗∗∗∗ p < 0.0001. (**I**) Correlations of the frequency of cells in the clusters with donor age. Dots–data from individual donors, line–simple linear regression. **C–E, G–I**) Each symbol represents data from an individual donor (n = 67 and 49 for the LF and HF groups, respectively); horizontal lines indicate group mean.

We initially quantified and phenotypically characterised NLV-T cells in PBMCs from 131 HLA-A^∗^02:01- and HCMV-positive individuals aged 22–88 years (Supplementary Table S1) using a spectral flow cytometry panel of 21 labelling reagents (Panel 1 in Supplementary Table S2 and Supplementary Fig. S1A). After excluding 15 samples that did not meet the quality control criteria (Fig. 1A), we found that the NLV-T cell frequencies ranged from <0.1% to >26% of all CD8 T cells (Fig. 1B and C). As frequencies of Epstein–Barr virus, influenza-, and SARS-CoV-2-specific CD8 T cells reach up to 1% of all CD8 T cells,^46^ we allocated the donors into LF (NLV-T cells <1%) and HF (NLV-T cells >1%) groups, to allow comparison of cells without and with NLV-T cell expansion.

For NLV-T cell detection, we used conventional tetramers (cTet). To gain simultaneous insight into the TCR avidity of NLV-T cells, we also co-stained cells with so-called null-tetramers (nTet), which bind TCRs independently of CD8 and thus mark T cells with high-avidity TCRs.^47^ While NLV-T cells bound both tetramers equally well, in approximately 35% of samples, we observed an NLV-T cell population binding nTet better than cTet, suggesting that it has higher avidity (Supplementary Fig. S1B–D). Additionally, in five samples, we also detected a third NLV-T cell population binding cTet better than nTet. However, the frequency of samples containing one, two, or three NLV-T cell populations did not differ between LF and HF donors (Supplementary Fig. S1C). All three populations were present in samples from LF and HF donors at comparable frequencies (Supplementary Fig. S1D). These findings are in agreement with previous reports,^24^, ^25^, ^26^ and suggest that NLV-T cells from LF and HF groups have comparable avidities.

There is a well-documented reversible correlation between the frequency of NLV-T cells and the expression of HLA-B^∗^07:02.^48^, ^49^, ^50^ To assure that HLA-B^∗^07:02 expression does not affect the distribution of donors into LF and HF groups, we compared NLV-T cell frequencies from 44 donors for whom HLA haplotypes were available. We found that distribution of donors into LF and HF groups or the frequency of NLV-T cells within the CD8 T cells is comparable between HLA-A^∗^02:01+HLA-B^∗^07:02- and HLA-A^∗^02:01+HLA-B^∗^07:02+ individuals (Supplementary Fig. S2). These data suggest that NLV-T cells can also expand in HLA-A^∗^02+HLA-B^∗^07+ donors.

The frequency of NLV-T cells positively correlated with their absolute number in the blood (Fig. 1D). On the other hand and consistent with previous findings,^10^^,^^28^^,^^31^ the frequency of NLV-T cells showed a weak correlation with donor age (Fig. 1E). Notably, NLV-T cell frequency was also independent of donor sex (Supplementary Fig. S3A). Together, these data suggest that the NLV-T cell expansion was a result of their proliferation and not due to age- or sex-related differences in white blood cell composition between donors.

Unsupervised clustering revealed that the majority of all NLV-T cells exhibited a TEMRA phenotype and could be further split into CD57^+^CD27^−^ and CD57^−^CD27^+^ subclusters (Fig. 1F). The third largest cluster contained cells with a Tem phenotype that also expressed CD27 and CD28. The fourth cluster contained NLV-T cells expressing CD45RA, CD45RO, and CD28, which we termed “mixed memory” cells. Finally, the smallest cluster contained NK-like NLV-T cells characterised by high expression of CD56. HF and LF donors contributed differently to the five clusters identified (Fig. 1G and H and Supplementary Fig. S3B). The LF donors had higher proportions of NLV-T cells showing Tem and mixed memory phenotypes, while the NLV-T cells from HF donors were more frequent in the two TEMRA clusters. The NLV-T cell distribution within clusters did not correlate with donor age (Fig. 1I), suggesting that subclinical HCMV reactivations, rather than donor age, could be the main factor driving the expansion of NLV-T cells with TEMRA phenotype.

Regardless of their phenotype, NLV-T cells expressed checkpoint molecules with no consistent differences between LF and HF donors (Supplementary Fig. S3C). These data are consistent with previous reports indicating varying expression of checkpoint molecules on circulating HCMV-specific T cells that is not related to T cell exhaustion.^5^ In conclusion, our comprehensive flow cytometry analysis revealed that all age groups of healthy donors contain individuals with expanded NLV-T cell populations that can be the best described as a mixture of Tem and TEMRA phenotypes.

### ScRNAseq of NLV-T cells from HF donors characterised them as GZMB^+^ effector/memory T phenotype

**Fig. 2:**
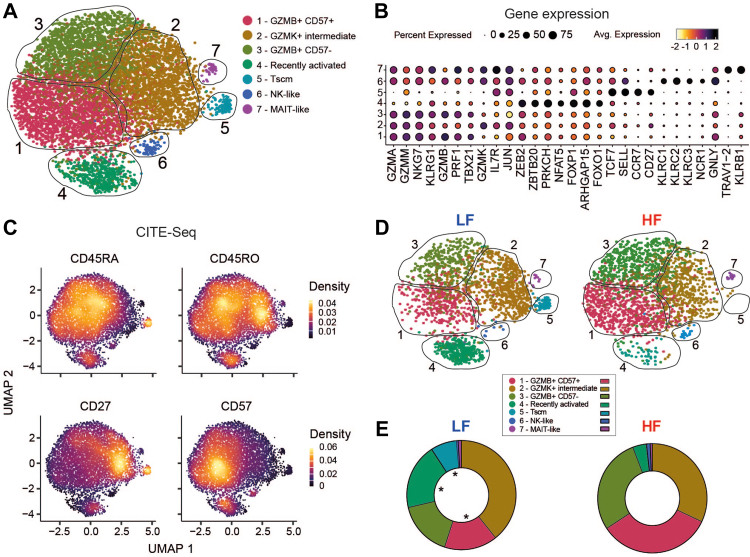
**HCMV-induced accumulation shifts the transcriptional landscape of NLV-T cells from the Tscm phenotype towards the GZMB^+^CD57^+^ effector/memory phenotype**. **A**) An UMAP plot showing cluster identities of individual NLV-T cells isolated from PBMCs of 8 LF donors and 12 HF donors. Each dot depicts one cell coloured according to the cluster identity determined by unsupervised clustering. **B**) A bubble plot indicating the expression of marker genes among different NLV-T cell clusters. Bubble size indicates the frequency of cells in a cluster expressing an individual gene. Bubble colour denotes the average gene expression. **C**) CITE-Seq-derived cell surface protein expression data overlaid on UMAP plot of NLV-T cells clustered by gene expression profiling. **D**) Downsampled UMAP plots split to show cluster identities from 1987 cells of LF and HF donors (n = 8 and 12, respectively). **E**) Doughnut plots showing NLV-T cell frequencies from LF and HF donors within different clusters. Two-way RM ANOVA (factors: donor group and cluster) followed by a two-stage linear step-up procedure of Benjamini, Krieger, and Yekutieli: ∗ p < 0.05.

To investigate the differences between NLV-T cells from LF and HF donors at a higher resolution, we next performed scRNAseq coupled with TCR profiling and cellular indexing of transcriptomes and epitopes by sequencing (CITE-Seq). As our phenotypic data indicated that age does not significantly affect NLV-T cell phenotype (Fig. 1I), we sorted NLV-T cells from randomly selected 8 LF (44.4 ± 16.2 years) and 12 HF donors (71.2 ± 3.6 years; Supplementary Fig. S4A). Unsupervised clustering organised 7376 NLV-T cells (1987 and 5389 from LF and HF groups, respectively) into seven clusters (Fig. 2A). The majority of cells were found in clusters 1–3 and expressed high levels of *GZMA, GZMM,* and *NKG7* and low levels of *CCR7* and *SELL*, identifying them as antigen-experienced effector/effector memory T cells. Cells in clusters 1 and 3 additionally expressed high levels of genes associated with effector T cells,^51^ such as *GZMB*, *KLRG1*, and *CX3CR1* (Fig. 2B and Supplementary Fig. S4B). NLV-T cells from clusters 1, 2, and 3 also expressed a mixture of genes recently described to distinguish Tem, TEMRA, and effector T cell (Teff) subpopulations (Supplementary Fig. S4C).^52^ Interestingly, cells in cluster 1 and 3 had a mixed CD45RA^+^ and CD45RO^+^ phenotypes but labelled differently with CITE-Seq antibody anti-CD57 (Fig. 2C). Therefore, we designated those clusters CD57^+^ (cluster 1) and CD57^−^ (cluster 3) granzyme B-expressing (GZMB^+^) effector T cells. We named cells in cluster 2 “granzyme K-expressing (GZMK^+^) intermediate differentiated T cells” as they expressed *GZMK* (but not *GZMB*) and preferentially labelled with CD45RO and CD27 CITE-Seq antibodies.^53^

NLV-T cells in clusters 4 and 5 expressed low levels of granzyme genes and *PRF1*. Cluster 4 NLV-T cells expressed activation (*ZEB2*, *ZBTB20*, or *NFAT5*) and memory markers (*FOXP1*, or *FOXO1*) (Fig. 2B), indicating that these cells might have been in recent contact with cognate antigen. Cluster 5 cells are characterised by high expression of *TCF7* (encoding for the transcription factor TCF1) and labelled with CD45RA and CD27 CITE-Seq antibodies (Fig. 2B and C). Thus, these NLV-T cells resemble the TCF1^+^ stem central memory T cell pool (TSCM), which has been suggested to maintain the inflationary T cell pool in mice infected with murine cytomegalovirus (MCMV).^54^ The two clusters containing the least number of cells expressed genes specific for natural killer (NK) cells (cluster 6: *KLRC1*, *KLRC2*, *NCR1*) or mucosal-associated invariant T cells (MAIT; cluster 7: *KLRB1*, *TRAV1–2*).^55^^,^^56^ Consequently, we designated NLV-T cells in clusters 6 as NK-like effector T cells and cells in cluster 7 as MAIT T cells.

Next, we examined the composition of NLV-T cell subsets in individual donor groups (Fig. 2D and E and Supplementary Fig. S4D). Compared to LF donors, the majority of NLV-T cells in HF donors had GZMB^+^CD57^+^ and GZMB^+^CD57^−^ phenotypes, indicating advanced differentiation. In contrast, a substantial proportion of NLV-T cells from LF donors showed recently activated or TSCM phenotypes, which were significantly reduced in HF donors. The intermediate GZMK^+^, NK-like, and MAIT-like NLV-T-cell subsets had comparable abundances in both LF and HF donors (Fig. 2D and E). To validate that observed differences are not influenced by the donor age, we stratified the donors into total of four subgroups: LF donors with 20–40 years of age (n = 4), LF donors with 55–65 years of age (n = 4), HF donors with 64–69 years of age (n = 4), and HF donors with 70–82 years of age (n = 8). Comparative analysis across these subgroups revealed that the differences in NLV-T cell subset distribution between LF and HF donors persist regardless of donor age (Supplementary Fig. 4E and F). Together, these data confirm that most NLV-T cells from HF donors are in an advanced state of differentiation, most accurately characterised as a CD57^±^GZMB^+^ effector/memory phenotype.

### NLV-T cell expansion induces donor-specific but phenotype-independent NLV-T cell clonality focussing

**Fig. 3:**
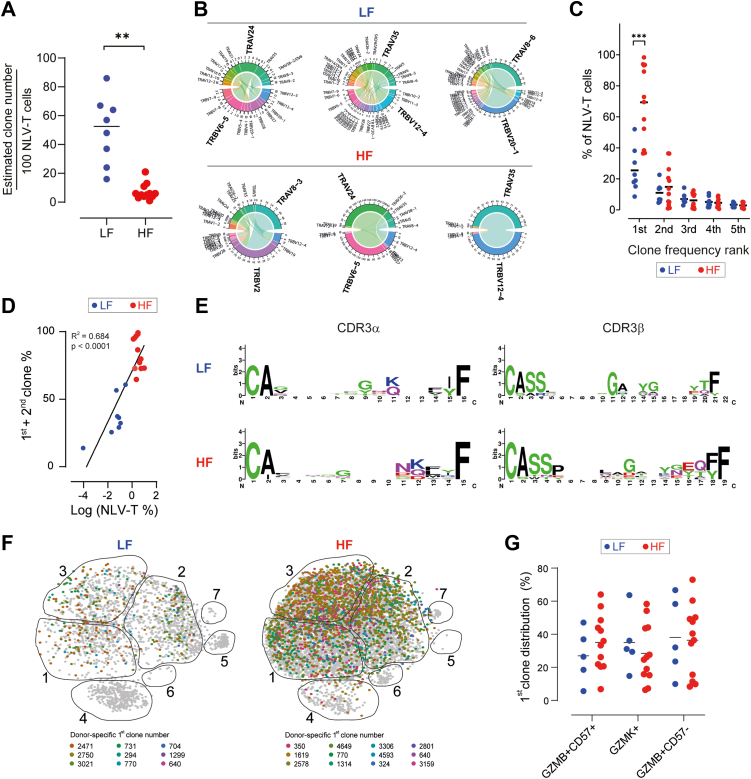
**HCMV-induced accumulation-induced focussing of donor-specific NLV-T cell clonality does not affect NLV-T cell phenotype**. **A**) NLV-T cell clonotype diversity expressed as the estimated number of unique clones per 100 cells. Unpaired t-test with Welch's correction: ∗∗ p = 0.0012. **B**) Circos plots depicting TRAV-TRBV gene pairing in NLV-T cells from three representative LF and HF donors. The top TRAV-TRBV pair in each donor is highlighted. **C**) Frequency of top five clones within total NLV-T cell number from each donor. Two-way ANOVA (factors: donor group and clone frequency rank) followed by Sidak's multiple comparisons test (LF *vs.* HF): ∗∗∗ p < 0.001. **D**) NLV-T cell frequency within the CD8 T cell gate correlates with the sum of the frequency of two top expanded clones. Dots–data from individual donors, line–simple linear regression. **E**) Representation of multiple sequence alignments for complementary determining region 3 of TCRα and TCRβ (CDR3α and CDR3β, respectively) in top NLV-T cell clones from LF and HF donors. **F**) UMAP plots from LF (left) and HF (right) donors depicting the NLV-T cells expressing the most frequent donor-specific clone. **G**) Distribution of donor-specific top expanded NLV-T cell clones within the three largest Seurat clusters. Two-way ANOVA (factors: donor group and cluster) followed by Sidak's multiple comparisons test (LF *vs.* HF): p > 0.05. **A, C, F**) Each symbol represents data from an individual donor (n = 8 and 12 for the LF and HF groups, respectively); horizontal lines indicate group mean.

We next analysed NLV-T cell clonality using paired TCR information from 5192 cells (943 and 4249 from LF and HF groups, respectively). NLV-T cells from LF donors contained approximately 7 times more unique clones than cells from HF donors (Fig. 3A and Supplementary Fig. S5A). The NLV-T cell clonal convergence was donor-specific and each donor had a personalised top-expanded combination of TRA variable (TRAV) and TRB variable (TRBV) gene segments (Fig. 3B and Supplementary Fig. S5B). Ranking of the top five NLV-T cell clones for each donor according to their relative population size revealed that NLV-T cell clonal convergence is more prominent in HF than LF donors (Fig. 3C). The comparison of LF and HF age subgroups indicated that differences in clonality between LF and HF donors are independent of the donor age (Supplementary Fig. S5C, D). Nevertheless, we observed significant variation in NLV-T cell clonal convergence among HF donors. In one-third of HF samples, the top expanded clone dominated, comprising ≥90% of the total NLV-T cell population. In contrast, in one-quarter of HF donors, it was similar in size to the second-largest NLV-T clone (Supplementary Fig. S5E). As the sum of the frequency of the top two expanded clones correlated with the total percentage of NLV-T cells within the CD8 gate (Fig. 3D), these findings suggest that NLV-T cell expansion primarily expands one or two donor-specific NLV-T cell clones. Interestingly, in two LF samples, the top two expanded clones occupied >50% of the total NLV-T cell number (Fig. 3C). These data suggest that clonal dominance is established even before NLV-T cell expansion becomes noticeable as a significant increase in NLV-T cell frequency.

The top expanded clones from each donor had little similarity in the complementary determining region 3 of the *TRAV* and *TRBV* genes (CDR3α and CDR3β, respectively; Fig. 3E). Furthermore, we found minimal overlap between *TRAV* and *TRBV* gene usage among NLV-T cells from both LF and HF donors and NLV-T cells from a publicly available dataset (Supplementary Fig. S5F).^57^ NLV-T cell clonotypes that express public TRAV-TRBV pairs were present in 8 out of 12 HF donors and 5 out of 8 LF donors. However, in 10 out of 13 donors, we found significantly less than 30 NLV-T cells expressing public TRAV-TRBV pairs per donor, which limited our ability to analyse these cells further. Taken together, these data suggest that latent HCMV infection favours the donor-specific expansion of CD8 T cell clones, consistent with previous findings with human CMV-specific T cells,^58^ and MCMV- and lymphocytic choriomeningitis virus (LCMV)-specific CD8 T cells in mice.^54^^,^^59^

To understand better how clonal dominance correlates to NLV-T cell phenotype, we examined distribution of NLV-T cells expressing the most expanded clone across the Seurat clusters. Surprisingly, the top NLV-T cell clones from all donors distributed through GZMK^+^, GZMB^+^CD57^−^, and GZMB^+^CD57^+^ clusters (Fig. 3F and G). These findings indicated that the expansion of NLV-T cells with the advanced differentiated phenotype in HF donors is not a result of a massive proliferation of a single NLV-T cell clone. In line with this hypothesis is also the observation that the number of clones within each of the three main clusters did not differ from the average estimated clone number of each group (Fig. 3A *vs.* Supplementary Fig. S5G). Moreover, no difference was observed between the LF and HF groups in the predicted TCR affinity scores of the top clones and the average affinity score of all NLV-T cell clones within each indicated cluster (Supplementary Fig. S5H, I).

### NLV-T cells proliferate *ex vivo* and acquire effector T cell phenotype

Our phenotypic and transcriptional analyses indicate that expansion drives the functional specialisation of NLV-T cells towards the GZMB^+^CD57^−^^/+^ phenotypes. Virus-specific cells with these phenotypes possess the most potent cytolytic effects.^7^^,^^60^^,^^61^

**Fig. 4:**
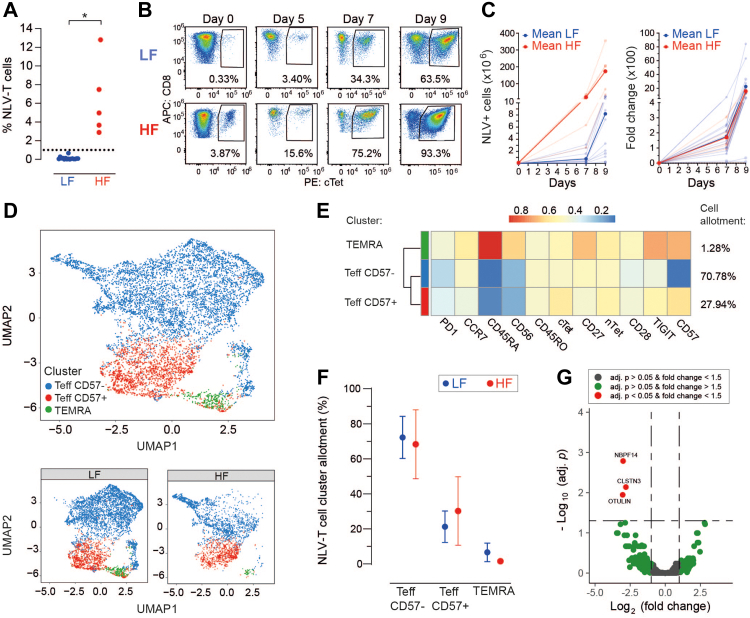
**NLV-T cells from LF and HF donors efficiently expand after peptide stimulation and acquire effector CD8 T cell phenotype (Teff)**. **A**) Frequency of NLV-T cells in selected samples before the expansion (please note that these data are also part of Fig. 1C). Each symbol represents data from an individual donor (n = 14 and 5 for LF and HF groups, respectively). Unpaired t-test with Welch's correction: ∗ p = 0.0251. **B**) Frequency of NLV-T cells within CD3+CD8+ cells before (day 0) and after peptide stimulation (day 5, 7, 9). Cells were labelled with Panel 1b antibodies from Supplementary Table S2 and gated as depicted in Supplementary Fig. S1A. Representative spectral flow cytometry data of one LF donor (top) and one HF donor (bottom). **C**) Expansion of NLV-T cells after peptide stimulation in total cell numbers (left) and fold change (right). Bold lines indicate the mean of the individual groups; thin lines indicate individual donors (n = 14 and 5 for LF and HF groups, respectively). **D, E**) Unsupervised clustering analysis of spectral flow cytometry data of expanded NLV-T cells (day 7) with grouping of cells into effector CD8 CD57^−^, effector CD8 CD57^+^, and TEMRA clusters. **D**) UMAP plots displaying NLV-T cells from all tested donors, as well as LF and HF groups. **E**) NLV-T cell marker expression heatmap. **F**) Subpopulation NLV-T cell frequencies. Data from 14 LF to 5 HF donors are presented as group mean (dots); error bars indicate standard deviation (SD). Two-way ANOVA (factors: donor group and cluster) followed by Sidak's multiple comparisons test (LF *vs.* HF): p > 0.05. **G**) Vulcano plot visualizing genes differently expressed between NLV-T cells from LF and HF donors at day 9 post-expansion. Adj. p–adjusted p value. Cut-off values, indicated with lines, for log2Fold change and adjusted p value were 1 and 0.05, respectively.

To examine NLV-T cell functions, we focused our analyses on 14 LF and 5 HF age-matched donors from the Transfusion Medicine cohort (Fig. 4A). These donors were matched by age, with a mean age of 46.6 ± 14.4 years for LF donors and 47.5 ± 9.3 years for HF donors. We have used only samples from this cohort in all subsequent experiments, as they provided the necessary cell numbers for NLV-T cell expansion. As we found no significant differences between cTet and nTet labelling (Supplementary Fig. S6A), we used only cTet labelling to follow NLV-T cells during expansion and found that the initial NLV-T cell frequency and phenotype did not affect peptide-mediated *ex vivo* NLV-T cell expansion (Fig. 4B and C). Unsupervised clustering analysis of expanded NLV-T cells showed a restricted phenotype compared to pre-expansion cells (Fig. 4D–F). Regardless of the donor of origin, expanded NLV-T cells exhibited either a CD57^+^ or CD57^−^ Teff phenotype, while only 1.28% of the NLV-T cells were found in the TEMRA cluster (Fig. 4E and F). Applying bulk RNA sequencing, we analysed transcriptomes of NLV-T cells and found minimal changes in the gene expression between NLV-T cells from LF and HF donors (Fig. 4G and Supplementary Fig. S6B). The genes significantly overexpressed in the cells from LF donors (neuroblastoma breakpoint family member 14, *NBPF14*; Calsyntenin-3, *CLSTN3*; and OTU deubiquitinase with linear linkage specificity, *OTULIN*) have no known role in T cell functions. Together, these data indicate that *ex vivo* expansion drives NLV-T cell differentiation into a Teff phenotype and that fast proliferation diminishes inter- and intra-individual phenotypical and transcriptional differences between NLV-T cells.

### Top expanded donor-specific NLV-T cell clones maintain their dominance after *ex vivo* proliferation

**Fig. 5:**
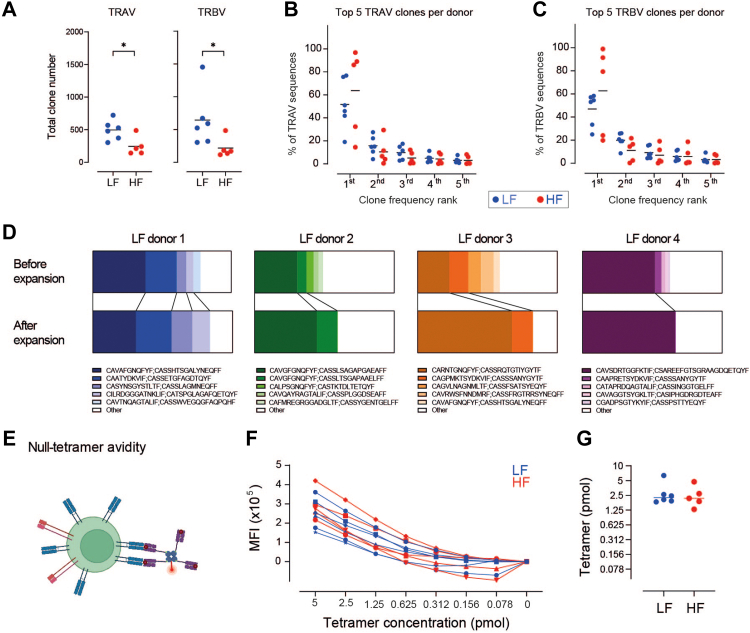
***Ex vivo* expanded NLV-T cells from LF and HF donors retain their donor-specific clonality and have comparable avidity and functional avidity**. PBMCs from 6 LF to 5 HF donors were stimulated with NLV peptide and cultured for 9 days to expand NLV-T cells. The cells were then stained with cTets, NLV-T cells were sorted, and TCR sequences were analysed using bulk RNA sequencing. **A**) TRAV and TRBV clonotype diversity of expanded NLV-T cells expressed as the estimated number of unique clones per 100 cells. Unpaired t-test with Welch’s correction: ∗ p < 0.05. **B, C**) Frequencies of top five TRAV (**B**) and TRBV (**C**) clones within total TRAV or TRBV clones from expanded NLV-T cells of each donor, respectively. Two-way ANOVA (factors: donor group and Clone frequency rank) followed by Sidak's multiple comparisons test (LF *vs.* HF): p > 0.05. **D**) Parts of whole stacked bar graphs showing the frequency of top 5 clones in the total TCR pool from 4 LF donors detected before and after 9 days of *ex vivo* NLV-T cell expansion after stimulation of donor PBMCs with the NLV-peptide. NLV-T cells before expansion were analysed using single-cell RNA sequencing and paired TRAV-TRBV gene information was used to define NLV-T cell clonality. The average expression of those *TRAV* and *TRBV* genes within bulk sequencing data after *ex vivo* expansion was used to determine the relative clone size after expansion. **E**) Scheme depicting NLV-T cell avidity determination using NLV-peptide loaded tetramers with mutated CD8 binding site (nTet). **F**) The median fluorescence intensity (MFI) values of NLV-nTet staining at indicated concentrations (each line represents data from one LF or HF donor). **G**) Calculated NLV-nTet concentration enabling half-maximal staining of expanded NLV-T cells (n = 6 and 5 for LF and HF groups, respectively). Unpaired t-test: p > 0.05. **A–C, G**) Each symbol represents data from an individual donor; horizontal lines indicate group mean.

Next, we determined whether *ex vivo* proliferation affects the clonality of NLV-T cells. Bulk RNA sequencing of samples from randomly selected six LF donors and five HF donors (42.5 ± 15.2 *vs.* 47.5 ± 9.4 years of age, respectively) revealed that NLV-T cells from HF donors retained restricted TRAV and TRBV clonality (Fig. 5A). Furthermore, donor-specific predominance of the top-expanded TRAV and TRBV clones was still present in all samples (Fig. 5B and C). To assess precisely the degree of changes induced by *ex vivo* proliferation on NLV-T cell clonality, we focused on the four LF donors whose samples were analysed before and after *ex vivo* expansion (Fig. 5D). As scRNAseq data provided the information on the combination of TRAV and TRBV clones for each NLV-T cell, we could extract the average expression of those TRAV and TRBV genes within bulk TCR sequencing data as a measure of the relative clone size after *ex vivo* proliferation. Importantly, we found that the top NLV-T cell clone retained its dominance in all four donors (Fig. 5D). In addition, in three out of four donors, the second most frequent clone was also present after *ex vivo* proliferation. Overall, these data demonstrate that *ex vivo* proliferation had a minimal impact on the NLV-T cell repertoire hierarchy.

Since the kinetics and magnitude of the T cell response depends on the affinity of TCR for its cognate antigen presented within the HLA molecules,^62^^,^^63^ we compared TCR avidities of the dominating NLV-T cell clones in our samples. Staining with decreasing concentrations of nTet revealed only minimal differences in TCR avidity between NLV-T cells from different donors within and between the two donor groups (Fig. 5E–G). These data are in line with our bioinformatically predicted TCR affinities between NLV-T cells from LF and HF donors before *ex vivo* proliferation (Supplementary Fig. S5F–G).

### *Ex vivo* expanded NLV-T cells from LF donors produce more TNF and IFNγ than NLV-T cells from HF donors

**Fig. 6:**
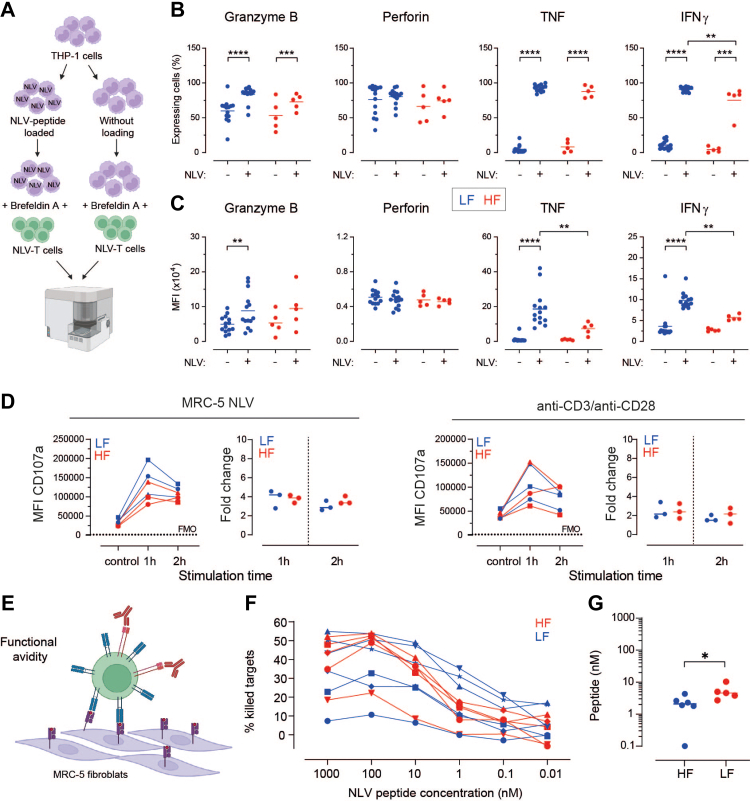
**Functional heterogeneity between *ex vivo* expanded NLV-T cells from LF and HF donors**. **A**) A THP-1 cell based stimulation assay scheme for determination of effector molecule expression in NLV-T cells after cognate antigen recognition. Please refer to the main text for details. **B, C**) Frequencies of NLV-T cells (**B**) and median fluorescent intensities (MFI) (**C**) of indicated effector molecules after overnight incubation with THP-1 target cells that were not loaded or loaded with NLV-peptide in the presence of Brefeldin A. (**B, C**) NLV-T cells are from 14 LF donors and 5 HF donors. Statistical analysis was performed using Two-way ANOVA (factors: donor group and NLV-peptide stimulation) followed by Sidak's multiple comparisons test: ∗∗ p < 0.01, ∗∗∗ p < 0.001, ∗∗∗∗ p < 0.0001. **D**) Degranulation assay based on flow cytometric analysis of CD107a expression on the NLV-T cells without (control) and after restimulation for 1 h or 2 h. *Ex vivo* expanded NLV-T cells from three randomly selected LF and HF donors were restimulated with MRC-5 cells loaded with 10 μM of the NLV-peptide (left set of graphs) or anti-CD3 and anti-CD28 antibodies (right set of graphs). Data are shown as MFI and fold change in CD107a MFI expression calculated for each donor relative to the MFI value of control staining. Fold change: Unpaired t-test with Welch's correction for each time-point: p > 0.05. **E**) Scheme depicting impedance-based killing assay using NLV-peptide loaded MRC-5 fibroblasts as targets to determine functional avidity of expanded NLV-T cells. Please refer to the main text for details. **F**) Plot depicting the percentage of killed target cells loaded with decreasing NLV-peptide concentrations and exposed to the NLV-T cells. Each line represents mean of 3–4 wells from individual LF or HF donor. **G**) Calculated peptide concentration enabling mean functional avidity (IC50) for NLV-T cells of each donor. Unpaired t-test: ∗ p < 0.05. (**F, G**) NLV-T cells are from 6 LF donors and 5 HF donors. (**B, C, D, G**) Each symbol represents data from an individual donor; horizontal lines indicate group mean.

To compare the functional capacity of NLV-T cells, we initially used intracellular flow cytometry to detect effector molecules within *ex vivo* proliferated NLV-T cells (Fig. 6A). As the target cells, we used THP-1 cells, a monocyte/macrophage cell line that also expresses HLA-A^∗^02:01 class I molecules. NLV-T cells from both LF and HF donors that were not in contact with cognate antigen had similar amounts of granzyme B and perforin and very low amounts, if any, of IFNγ and (Fig. 6B and C, and Supplementary Fig. S7A). On the other hand, recognition of the NLV peptide loaded on target cells increased the percentage of cytokine-expressing NLV-T cells from both LF and HF donors (Fig. 6B). Comparison of the MFI of the cytokine labelling per cell revealed that NLV-T cells from LF donors produced 1.72- and 2.52-fold more TNF and IFNγ, respectively than the cells from HF donors (Fig. 6C). Similarly, antigen-recognition increased the amount of granzyme B within the NLV-T cells from both LF and HF donors (Fig. 6B and C). On the other hand, the NLV-mediated stimulation of NLV-T cells from HF donors did not affect their perforin content (Fig. 6B and C). A more detailed analysis of perforin-positive NLV-T cells revealed an increased frequency of cells producing high levels of cytokines in the samples from LF than in HF donors (Supplementary Fig. S7B, C). These data indicate that although NLV-T cells are multifunctional, a certain degree of functional heterogeneity exists between NLV-T cells from LF and HF donors.

To compare the NLV-T cell capacity to degranulate upon antigen encounter, we measured cell surface modulation of CD107a upon activation with anti-CD28/-CD3 antibodies or NLV-peptide-loaded adherent HLA-A^∗^02:01+ cell line, MRC-5 fibroblasts. The kinetic and extent of granule release, measured using *ex vivo* expanded cells of three randomly selected donors from each group, revealed that NLV-T cells from LF and HF donors had comparable levels of degranulation (Fig. 6D).

We next investigated functional avidity of NLV-T cells in the impedance-based killing assay. For this assay we loaded MRC-5 fibroblasts with decreasing doses of NLV peptide (Fig. 6E and Supplementary Fig. S7D). As expected, the killing efficacy of NLV-T cells correlated with the peptide concentration (Fig. 6F). We observed variation in maximal killing efficacy between NLV-T cells from different donors, which was not significantly different between the LF and HF groups (Supplementary Fig. S7E). However, NLV-T cells from HF donors required on average 2.7-fold higher peptide concentration for 50% maximum target cell lysis than those from LF donors (5.88 ± 3.51 nM *vs*. 2.17 ± 1.36 nM; Fig. 6G). Altogether, these data suggest that NLV-T cells from LF donors are more effective at eliminating infected cells that present limited amounts of antigen, such as those that have undergone HCMV-induced downregulation of HLA type I molecules.

### *Ex vivo* expanded NLV-T cells from LF but not HF donors eliminate HCMV-infected macrophages

**Fig. 7:**
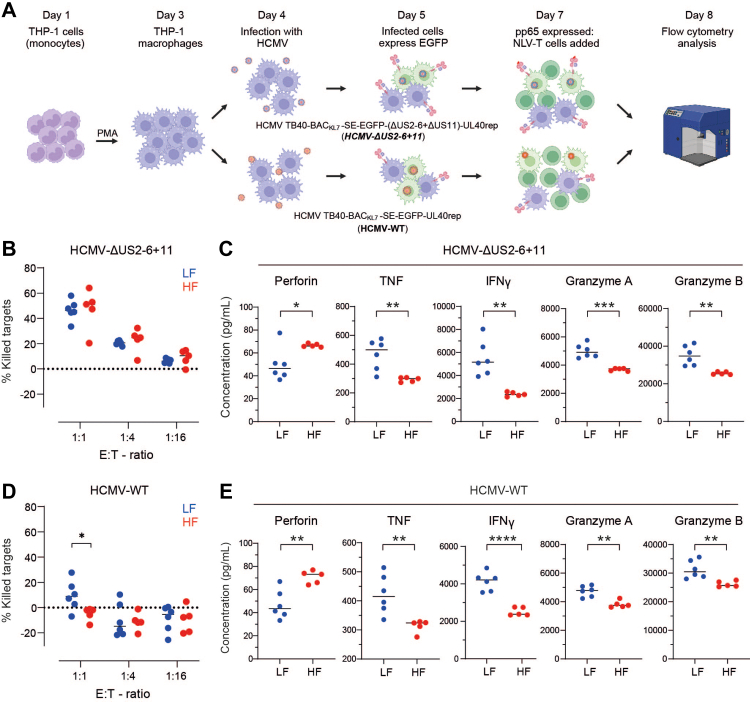
**NLV-T cells from LF but not HF donors kill HCMV-infected macrophages despite virus-induced HLA downregulation**. **A**) Overview of the *in vitro* killing assay using TB40-BAC_KL7_-SE-EGFP-UL40_rep_ (HCMV-WT) and HCMV TB40-BAC_KL7_-SE-EGFP-(ΔUS2-6+ΔUS11)-UL40_rep_ (HCMV-ΔUS2-6+11) infection of THP-1 macrophages. PMA: phorbol 12-myristate 13-acetate. **B**) NLV-T cell killing efficacy of HCMV-ΔUS2-6+11-infected target cells at indicated effector to target (E:T) cell ratios. Two-way ANOVA (factors: donor group and E:T ratio) followed by Sidak's multiple comparisons test (LF *vs.* HF): p > 0.05. **C**) Concentrations of indicated effector molecules in the supernatants collected from the killing assay of HCMV-ΔUS2-6+11-infected target cells with E:T 1:1. Unpaired t-test with Welch's correction: ∗ p < 0.05, ∗∗ p < 0.01. **D**) NLV-T cell killing efficacy of HCMV-WT-infected target cells at indicated E:T cell ratios. Two-way ANOVA (factors: donor group and E:T ratio) followed by Sidak's multiple comparisons test (LF *vs.* HF): ∗ p < 0.05. **E**) Concentrations of indicated effector molecules in the supernatants collected from the killing assay of HCMV-WT-infected target cells with E:T 1:1. Unpaired t-test with Welch's correction: ∗ p < 0.05, ∗∗ p < 0.01. (**B–E**) Each symbol represents data from an individual donor (n = 6 and 5 for LF and HF groups, respectively); horizontal lines indicate group mean.

To investigate NLV-T cell killing efficacies towards HCMV-infected cells, we utilised two reporter HCMV strains. The HCMV variant TB40-BAC_KL7_-SE-EGFP-UL40_rep_ (HCMV-WT) is clinically relevant reporter virus strain.^34^ It encodes an enhanced green fluorescent protein (EGFP) under the control of the viral major immediate early promoter, enabling easy detection of infected cells using flow cytometry. The second virus strain was an HCMV mutant variant (HCMV TB40-BAC_KL7_-SE-EGFP-(ΔUS2-6+ΔUS11)-UL40_rep_; HCMV-ΔUS2-6+11) lacking the immune-evasion genes US2, US3, US6, and US11.^37^ Therefore, this virus strain is unable to downregulate HLA class I molecules on the surface of the infected cells (Supplementary Fig. S8A). We used these two HCMV reporter viral strains to infect macrophages derived from PMA-differentiated THP-1 cells (Fig. 7A). At 3 days post-infection, the time-point of peak pp65 protein expression,^64^ we added *ex vivo* expanded NLV-T cells. After 17–22 h of co-culture, we quantified EGFP-expressing infected cells by flow cytometry (Supplementary Fig. S8B). With this setup, we kept the total culture period shorter than the 96 h required for HCMV to finish the lytic replication cycle in fibroblasts,^65^ ensuring that the number of HCMV-infected cells remained comparable to that of non-infected polarised macrophages (Supplementary Fig. S8C).

While NLV-T cells from both donor groups efficiently eliminated HCMV-ΔUS2-6+11-infected targets (Fig. 7B), they differed significantly in their production of effector molecules (Fig. 7C). Cells from HF donors produced higher amounts of perforin while the cells from LF donors released larger quantities of TNF and IFNγ. Interestingly, we also found higher concentrations of granzymes A and B in the supernatants of the target cells co-cultured with NLV-T cells from LF donors (Fig. 7C). Other analysed effector molecules were released at comparable levels (Supplementary Fig. S8D).

HLA downregulation allowed the HCMV-WT strain to escape recognition by NLV-T cells from both donor groups to various degrees (Fig. 7D). NLV-T cells from HF donors were ineffective in eliminating HCMV-WT-infected targets, while NLV-T cells from LF donors at an E:T ratio of 1:1 still managed to reduce the number of infected cells by 9.6% ± 11.9% (Fig. 7D). Interestingly, effector-molecule production of NLV-T cells from LF and HF donors matched the pattern found in cultures with HCMV-ΔUS2-6+11-infected targets (Fig. 7E and Supplementary Fig. S8E). Nevertheless, only in supernatants from co-cultures of NLV-T cells with targets infected with the HCMV-WT strain the concentrations of perforin reversely correlated to specific lysis, while TNF and IFNγ concentrations positively correlated by trend to specific lysis (Supplementary Fig. S8F).

## Discussion

Here, we describe differentiation-induced reduction in functional CD8 T cell diversity as a novel mechanism that allows HCMV to escape immune protection. Through a comprehensive comparison of NLV-T cells, we discovered distinct patterns of NLV-T cell differentiation that impact their ability to clear HCMV-infected cells. NLV-T cells from LF donors had a less-differentiated GZMK^+^ phenotype, were multifunctional, and were even capable of killing HCMV-infected cells with virus-induced downregulation of HLA surface protein. The NLV-T cell expansion tipped the balance towards a more terminally differentiated CD57^+^GZMB^+^ effector/memory phenotype. Expanded NLV-T cells retained their multifunctionality but produced lower amounts of cytokines upon cognate antigen recognition. These cells were still potent in killing targets presenting high levels of cognate antigen but failed to destroy HCMV-infected cells with virus-induced suppression of HLA expression. Taken together, our findings offer a potential explanation for the commonly observed weakening of HCMV reactivation control in individuals with NLV-specific T cell expansion.^9^, ^10^, ^11^, ^12^

Observed shift from NLV-T cells with GZMK^+^ intermediate phenotype in LF donors towards more differentiated GZMB^+^ NLV-T cells in HF donors aligns with reports indicating considerable heterogeneity in non-latent pathogen-specific CD8 T cells.^66^^,^^67^ Recently, GZMK^+^ CD8 T cells were described as a core cytokine-producing population across different human tissues and diseases.^68^ Repetitive antigen stimulation with non-latent pathogens induces gradual CD8 T cell differentiation,^69^^,^^70^ shifting them towards TEMRA phenotypes^69^^,^^71^ with increased GZMB expression.^72^^,^^73^ Advanced stage differentiation of CD8 T cells is also characterised by increased perforin expression,^53^ which is in line with our findings of increased perforin production from NLV-T cells from HF donors during elimination of HCMV-infected targets. It is therefore intriguing to speculate that the observed differentiation of NLV-T cells reflects repetitive antigen stimulation due to HCMV subclinical reactivations.

Despite distinct phenotype characteristics, NLV-T cells from LF and HF donors had comparable TCR avidity. These data are in line with previous reports.^24^, ^25^, ^26^ In contrast, CD8 T cells specific for IE-1 protein undergo reverse TCR repertoire evolution, resulting in expansion of HCMV-specific CD8 T cells with low-affinity T cell receptors.^19^ These divergence in TCR affinities suggests that IE-1-specific and pp65-specific CD8 T cells follow distinct pathways, most likely reflecting the frequency of antigen restimulation.^74^ During latency, HCMV periodically expresses a limited set of proteins, including IE-1.^75^^,^^76^ This permanent viral antigen source could induce proliferative senescence of high-avidity IE-1-specific T cells, eventually replacing them with low-affinity clones, as proposed for CD8 T cells specific for another persistent herpesvirus, the Epstein–Barr virus (EBV).^77^

Our data support a model where viral transcripts expressed during HCMV replication selectively expand high-avidity T cells.^74^ We observed that the top TCR clones from HF donors did not show uniform phenotypes but represented a mixture of less-differentiated GZMK^+^ intermediates and also more differentiated GZMB^+^ phenotypes. These findings might reflect a history of antigen encounters for each NLV-T cell. As these cells patrol the body to control for HCMV reactivation,^6^ some cells likely encounter viral antigens more frequently and differentiate into a more advanced GZMB^+^CD57^+^ phenotype. This hypothesis also explains why some NLV-T cells from LF donors already acquired the GZMB^+^CD57^+^ phenotype, while NLV-T cells that do not frequently encounter their cognate antigen might retain the GZMK^+^ intermediate phenotype. Moreover, the same hypothesis could explain the expansion of NLV-T cells in HF donors. In individuals with a history of frequent HCMV reactivations, such as older persons infected early in life, NLV-T cells may encounter the antigen more frequently, leading not only to differentiation but also to their proliferation. Consequently, NLV-T cells with a GZMB^+^CD57^+^ phenotype would accumulate, which might serve as a mechanism to compensate for the lack of function in these cells. Therefore, our data suggest that a donor-specific history of HCMV-reactivation affects the NLV-T cell number and shapes overall balance of their populations between less and more differentiated phenotypes.

We also found that the transition from a less to a more differentiated phenotype impacts the functional properties of NLV-T cells. Our data showing that NLV-T cells from LF and HF donors express different levels of effector molecules extend previous observations reporting that the expansion of NLV-T cells is accompanied by decreased frequency of antigen-specific IFNγ- and IL-10-producing cells^29^^,^^31^^,^^32^ and increased granzyme B and perforin expression.^78^ Moreover, HCMV-specific CD57^−^ TEMRA cells have higher HCMV peptide sensitivity in an IFN-γ release assay compared to their CD57^+^ counterparts.^61^ Thus, it is intriguing to speculate that higher levels of cytokine production offer a competitive advantage for NLV-T cells from LF donors to eliminate targets presenting minuscule amounts of antigen. TNF might directly kill target cells,^79^ and IFNγ increases antigen presentation by stimulating HLA expression.^80^ The later might be particularly important, as the downregulation of surface HLA molecules impairs CD8 T cell ability to kill its targets.^81^ Additionally, TNF and IFNγ have also direct antiviral effects, such as inhibition of HCMV replication and reduction in the amount of infectious virus.^82^, ^83^, ^84^ As NLV-T cells from HF donors still produce substantial amounts of these cytokines, it remains to be determined whether they can still impact HCMV replication.

We have also observed additional differences between NLV-T cells from LF and HF donors that could influence their functional properties, such as divergent perforin and granzymes A and B concentrations in killing assays on HCMV-infected macrophages. Moreover, our transcriptional profiling indicated differences in the expression of other granzymes between NLV-T cells with GZMK^+^ intermediate and GZMB^+^ phenotypes. The CD8 T cell differentiation status affects the profile of granzyme expression,^53^ and the individual contribution of each granzyme to killing depends on the target cell itself.^85^ Thus, differences in granzyme molecule expression rather than in cytokine secretion may affect the killing of NLV-T cell targets. Moreover, NLV-T cells with a GZMB^+^ phenotype expressed also higher levels of natural killer cell granule protein-7 (NKG7). Among other roles, NKG7 enhances CD8 T cell synapse efficiency and, thus, CD8 T cell killing of tumour targets.^86^ Conversely, it is possible that increased NKG7 expression in HF donor NLV-T cells prevented them from establishing stable synapses on the targets presenting limited amounts of antigen. Interestingly, cytokine hypersecretion enables NKG7-deficient CD8 T cells to eliminate their tumour targets.^86^ Therefore, future experiments investigating the killing mechanisms of NLV-T cells should involve a detailed examination of their interactions with target cells.

Our finding that NLV-T cells from HF donors cannot kill HCMV-infected targets with virus-induced downregulated HLA molecules has several important implications. In otherwise healthy individuals with NLV-T cell expansion, differentiation-induced weakening of NLV-T protective functions offers an elegant explanation for the observed increase of HCMV reactivation in individuals with expanded HCMV-specific T cell populations.^9^, ^10^, ^11^, ^12^ Furthermore, as pp65 is one of the standard target antigens for immunotherapy of transplant-associated HCMV reactivation,^87^^,^^88^ our results should also be considered when selecting the donors for adoptive antiviral T cell immunotherapy. Our data suggest that cell products generated from LF donors could offer better protection than those from donors with expanded NLV-T cell populations. We previously reported that 50% of the donors had high frequencies of pp65-specific T cells in the CD45RA- T cell fraction,^89^ indicating that products from those donors would contain cells that could efficiently kill virus-infected cells. Interestingly, Mülling et al. recently described that kidney transplant patients who developed HCMV viraemia had lower frequencies of IFNγ producing NLV-T cells than their counterparts from patients who successfully controlled HCMV infection.^90^ In that study, metabolic changes in NLV-T cells impaired the control of the CMV infection, and inhibition of the enzymatic activity of the ectoenzyme CD38 improved NLV-T cell function.^90^ Hence, future studies should investigate whether there are differences in the metabolism of NLV-T cells from LF and HF donors.

Our findings also prompt the question of whether other HCMV-specific T cells can compensate for the diminished functionality of NLV-T cells in HF donors. Our data suggest that some HF donors possess the HLA-A^∗^02+ HLA-B^∗^07+ haplotype. HCMV-specific cellular immune responses to pp65 restricted by HLA-B∗07 dominate HLA-A∗02 responses,^48^, ^49^, ^50^ raising the possibility that in these individuals, pp65-specific HLA-B∗07-restricted CD8 T cells may protect against latent HCMV infection. Furthermore, NLV-T cells may have been replaced by CD8 T cells specific to other HCMV proteins, such those specific to IE-1 protein. Therefore, future studies should carefully investigate the functionality of NLV-T cells in HF and LF donors in the context of the donor HLA haplotype and all other HCMV-specific T cells.

In conclusion, we provided a comprehensive phenotypical and functional characterisation of virus-specific T cells in healthy HCMV-seropositive donors. Our analyses revealed significant differences in effector molecule production and function between non-expanded NLV-T cells and their counterparts that expanded during latent HCMV infection. NLV-T cell expansion led to a reduction in IFNγ and TNF production, impairing the ability of NLV-T cells to eliminate HCMV-infected cells with virus-induced downregulated surface expression of HLA molecules. Our findings demonstrate that expansion significantly alters NLV-T cell functionality, thus identifying a previously unrecognised mechanism of HCMV immune evasion, in which differentiation of CD8 T cells compromises their ability to protect against the virus effectively. These data have significant implications for various areas, including immunosenescence, adoptive T cell transfer, and using HCMV as a potential vaccine vector.

### Limitations of the study

The exact mechanisms of the expansion-induced NLV-T cell differentiation and the significance of reduced cytokine production on the killing mechanisms remain to be determined. Furthermore, the described differentiation-induced loss of protective functions might not apply to CD8 T cells specific to other HCMV proteins or pp65-antigens presented in other HLA alleles.

## Contributors

L.F. and B.B. conceptualised the work and designed the experiments. L.F. phenotypically characterised NLV-T cells, expanded them, and conducted all functional experiments. L.R. conducted unsupervised clustering of flow cytometry data. A.H. and H.G. performed single-cell RNA and bulk sequencing experiments, and A.H. and B.B. analysed the data. L.F., A.H., and B.B. have directly accessed and verified the underlying data. B.C. and B.Č. produced the HCMV stocks and B.W. provided support during impedance experiments. B.E.-V., L.M.R., T.W., C.F., and R.F. provided the donor samples and/or contributed with resources. R.F. and B.B. jointly supervised the work. L.F. and B.B. wrote the first manuscript draft. All authors contributed to the manuscript draft correction and approved the submitted version.

## Data sharing statement

The generated single-cell RNA and bulk sequencing data have been submitted to the National Centre for Biotechnology Information/Gene Expression Omnibus database (https://www.ncbi.nlm.nih.gov/geo) under accession number GSE289829. All data are available in the main text or the Supplementary Materials.

## Declaration of interests

The authors have no conflicting financial interests.
