# Supplementary material for: Differentiation-induced reduction in functional diversity restricts the ability of cytomegalovirus-specific CD8 T cells to eliminate virus-infected cells
Source: eBioMedicine. 2026 Jan 5;123:106107. doi: 10.1016/j.ebiom.2025.106107 (PMC12809745; doi:10.1016/j.ebiom.2025.106107)
Supplement: Supplementary Figures and Tables [file mmc1.docx]

**Differentiation-induced reduction in functional diversity restricts the ability of cytomegalovirus-specific CD8 T cells to eliminate virus-infected cells**

Dr. Lea Fritz^1^, Dr. Ahmed Hassan^1^, Dr. Lennart Riemann^1,2^, Berislav Čuvalo^1^, Dr. Bibiana Costa^3^, Britta Wieland^4^, Prof. Britta Eiz-Vesper^5,6^, Prof. Christine Falk^7,8^, Dr. Lennart M. Roesner^9,10^, Prof. Thomas Werfel^9,10^, Prof. Ulrich Kalinke^3,10^, Dr. Hristo Georgiev^1^, Prof. Reinhold Förster^1,6,10,†^, Dr. Berislav Bošnjak^1,10*†^

^1^ Institute of Immunology, Hannover Medical School, Hannover, Germany

^2^ Department for Paediatric Pneumology, Allergology and Neonatology, Hannover Medical School, Hannover, Germany

^3^ Institute for Experimental Infection Research, TWINCORE, Centre for Experimental and Clinical Infection Research, a joint venture between the Helmholtz Centre for Infection Research and the Hannover Medical School, Hannover, Germany

^4^ University Women's Hospital, Hannover Medical School, Hannover, Germany

^5^ Institute of Transfusion Medicine and Transplant Engineering, Hannover Medical School, Hannover, Germany

^6^ German Centre for Infection Research (DZIF), Partner Site Hannover-Braunschweig, Hannover, Germany

^7^ Institute of Transplantation Immunology, Hannover Medical School, Hannover, Germany

^8^ German Centre for Lung Research (DZL), BREATH Site, Hannover, Germany,

^9^ Department of Dermatology and Allergy, Hannover Medical School (MHH), Hannover, Germany

^10^ Cluster of Excellence RESIST (EXC 2155), Hannover Medical School, Hannover, Germany

^†^ These authors contributed equally to this work.

* Corresponding author:

Dr. Berislav Bošnjak, PD, Institute of Immunology, Hannover Medical School, Carl-Neuberg Straße 1, 30625 Hannover, Germany, phone: +49 511 532 9729, email: bosnjak.berislav@mh-hannover.de


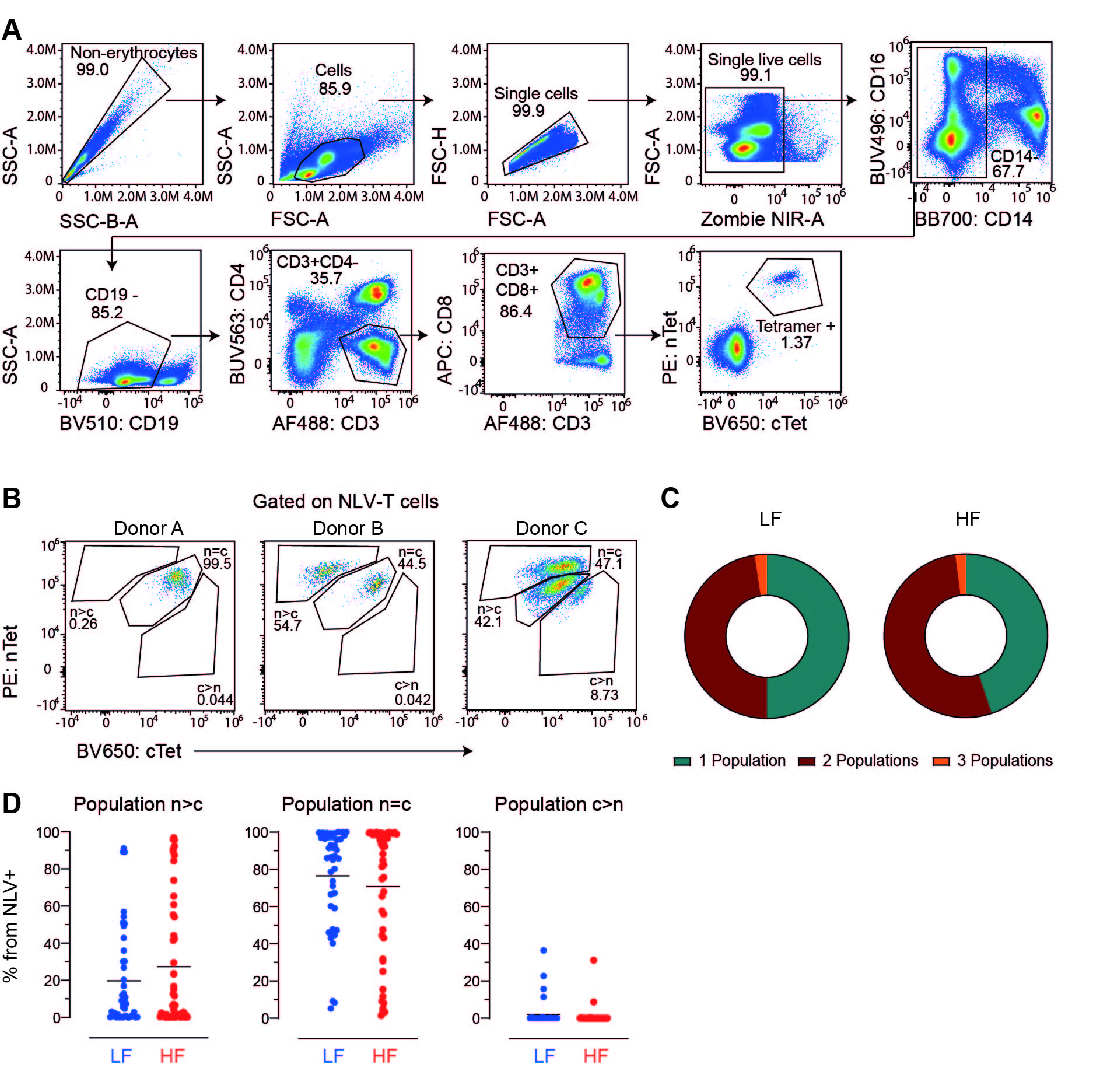


**Fig. S1.** **Spectral flow cytometry analysis of NLV-T cells, related to Fig. 1.**

**A**) Gating strategy for the NLV-T cell analysis. **B**) Staining of Tetramer positive cells gated as in A with conventional tetramers (cTet) and so-called null-tetramers (nTet) distinguished three NLV-T cell populations. Dot plots depicting NLV-T cells from representative donors with either one (left), two (middle), or three (right) populations, defined according to cTet and nTet staining as: n>c (P1), n=c (P2), or c>n (P3). **C**) NLV-T cell distribution into populations 1-3 does not differ between LF and HF donors. **D**) Distribution of LF and HF donors according to the number of NLV-Tetramer-positive populations detected with cTet and nTet staining. Each symbol represents data from an individual donor; horizontal lines indicate group mean. Unpaired t test with Welch’s correction, p > 0.05. (**C,D**) n=67 and 49 for the LF and HF groups, respectively.


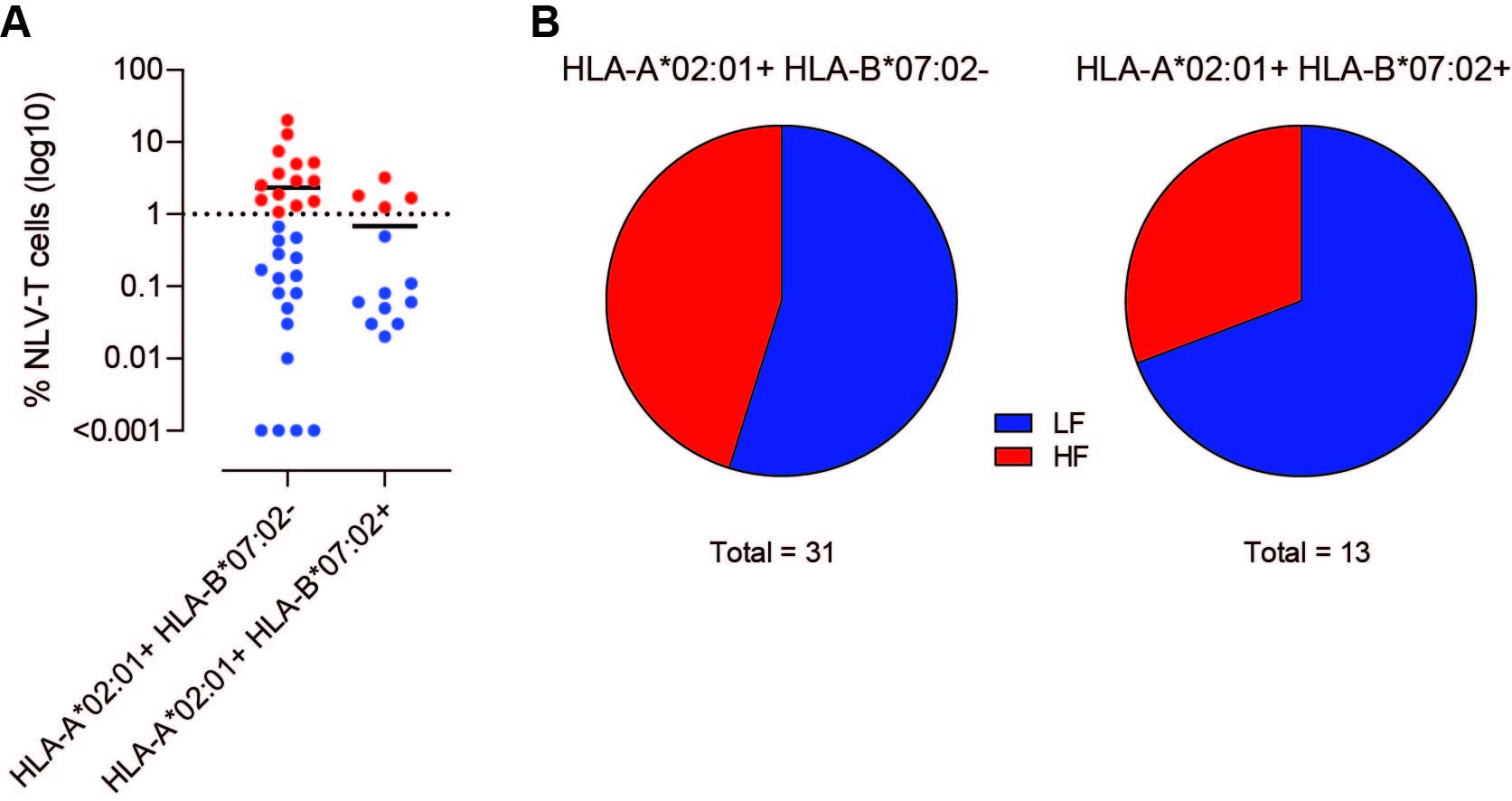


**Fig. S2.** **HLA-B*07:02 expression does not affect the distribution of donors into LF and HF groups, related to Fig. 1.**

**A**) The NLV-T cell frequency within the CD3+CD8+ T cell gate, HLA-A*02:01+HLA-B*07:02- and HLA-A*02:01+HLA-B*07:02+ donors were split into high-frequency (NLV-T cells > 1%, HF) and low-frequency (NLV-T cells < 1%, LF) groups. Unpaired t-test with Welch’s correction on log-transformed NLV-T cell frequencies, p > 0.05. **B**) Distribution of HLA-A*02:01+HLA-B*07:02- and HLA-A*02:01+HLA-B*07:02+ individuals into LF and HF groups. Fisher’s exact test, p > 0.05. (**A,B**) HLA-A*02:01+HLA-B*07:02-: 17 LF donors and 14 HF donors, HLA-A*02:01+HLA-B*07:02+: 9 LF donors and 4 HF donors.


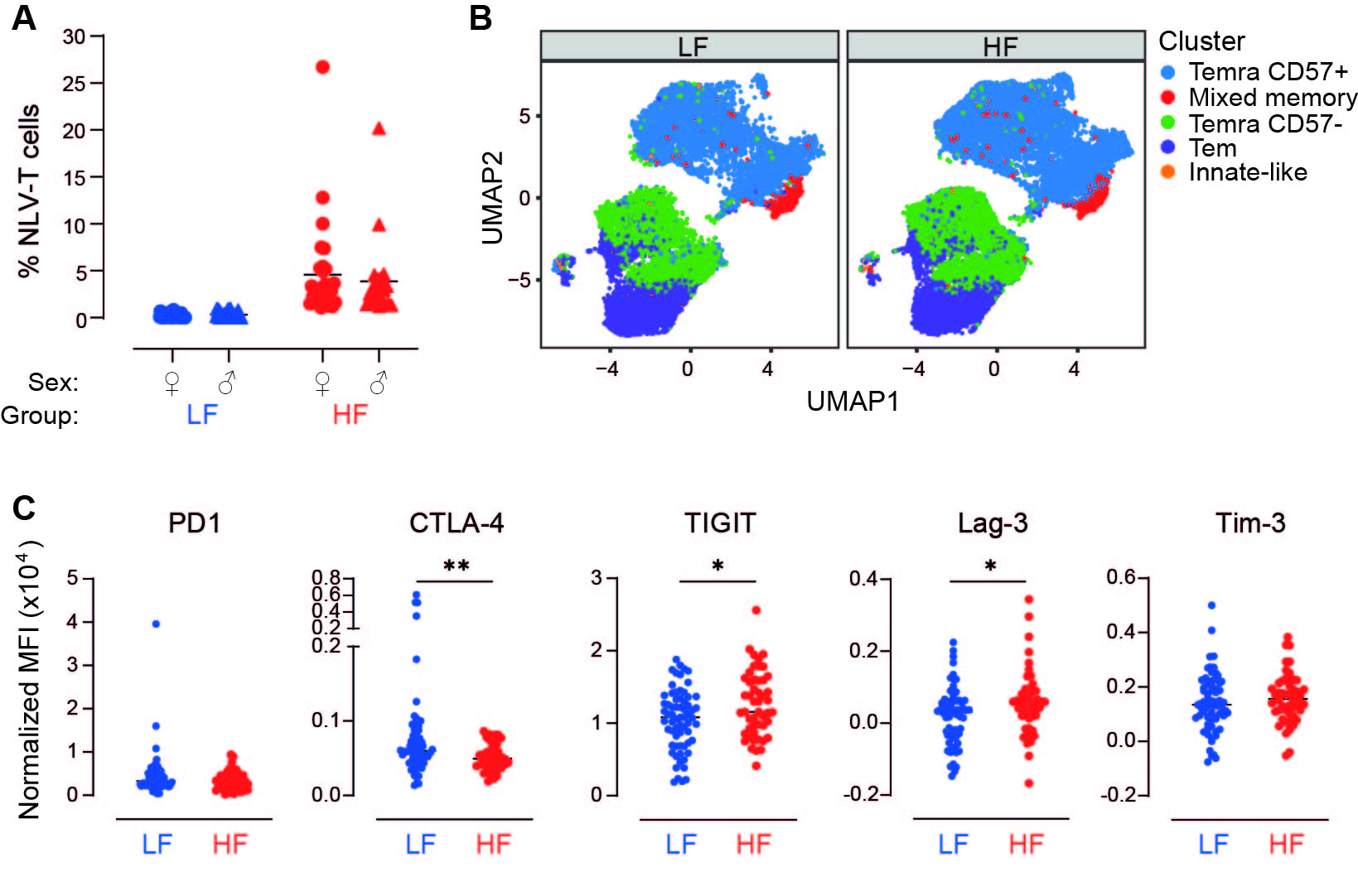


**Fig. S3: Additional NLV-T cell characterization, related to Fig. 1.**

**A**) The NLV-T cell percentage within the CD3+CD8+ T cell gate is not affected by donor sex between or within both investigated groups. Welch’s ANOVA test followed by Dunnett’s T3 multiple comparison test, LF male *vs.* LF female and HF male *vs.* HF female, p > 0.05. **B**) NLV-T cell composition presented as UMAP plots generated from concatenated NLV-T cells from LF donors (left) and HF donors (right). Cluster designation as in Fig. 1f. **C**) Expression of the checkpoint molecules PD-1, CTLA-4, TIGIT, Lag-3, and Tim-3 on NLV-T cells from LF and HF donors. Unpaired t-test with Welch’s correction on transformed data (Y=Y/K; K=10000): * p < 0.05, ** p < 0.01. **A, C**) Each symbol represents data from an individual donor (n=67 and 49 for the LF and HF groups, respectively); horizontal lines indicate group mean.


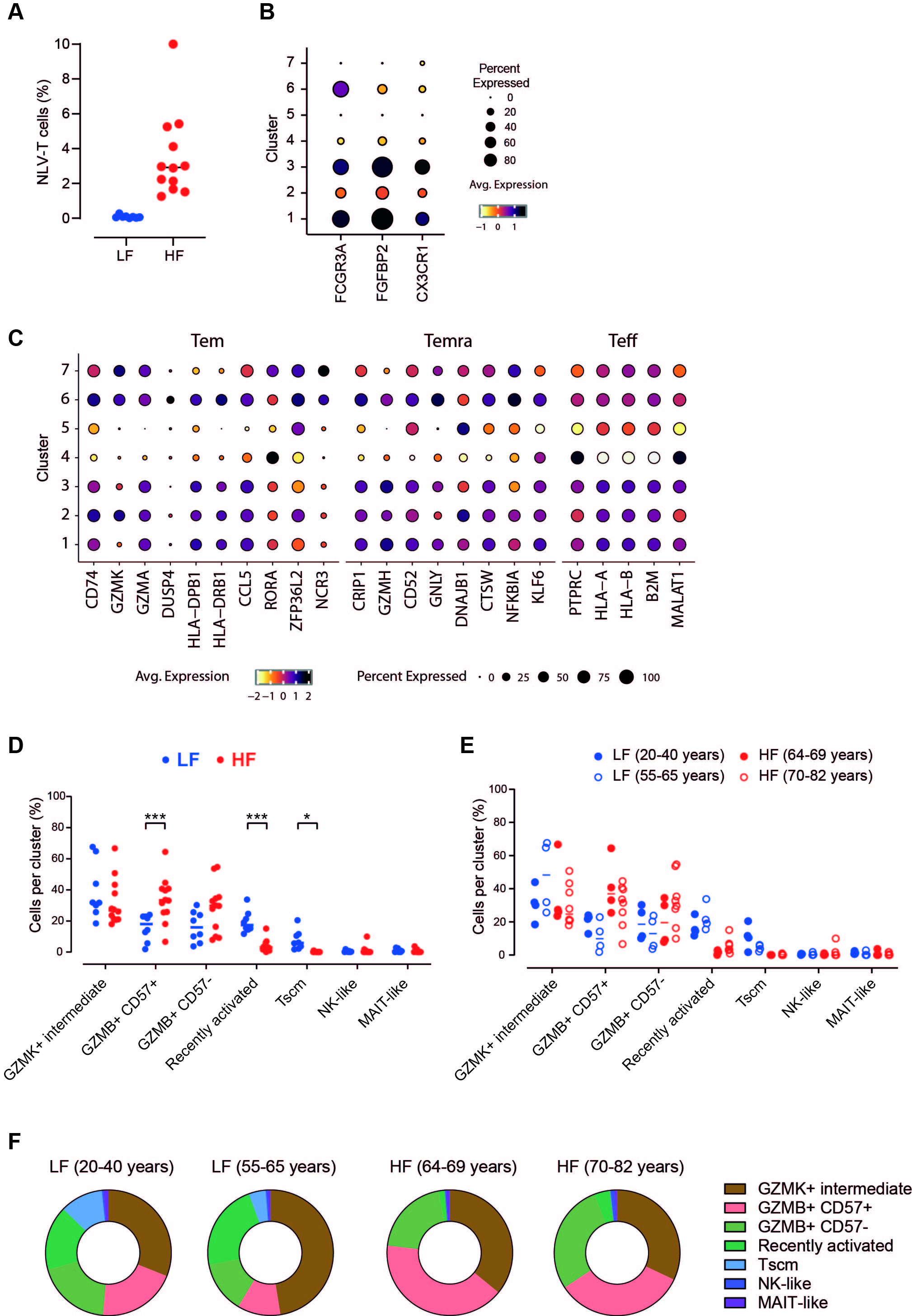


**Fig. S4: Additional information for single-cell RNA sequencing of NLV-T cells from low-frequency (LF) and high-frequency (HF) donors, related to Fig. 2.**

**A**) Frequency of NLV-T cells in 8 LF and 12 HF donors selected for single-cell RNA sequencing (please note that these data are part of Fig. 1c). **B, C**) A bubble plots indicating gene expression in NLV-T cell clusters numbered according to Figure 2a. Genes were chosen according to (**B**) [51] or (**C**) [52]. Bubble size indicates the frequency of cells in a cluster expressing an individual gene. Bubble color denotes the average gene expression. **D**) Distribution of NLV-T cells from individual LF and HF donors in different clusters, designated according to Fig. 2A. Two-way RM ANOVA (factors: Donor group and Cluster) followed by a two-stage linear step-up procedure of Benjamini, Krieger, and Yekutieli: * p < 0.05; *** p < 0.001. **E**) Cluster distribution of NLV-T cells from individual LF and HF donors stratified by age. **F**) Donut plots showing NLV-T cell frequencies from LF and HF donors within different clusters. **A, D**) Each symbol represents data from an individual donor (n= 8 and 12 for the LF and HF groups, respectively); horizontal lines indicate group mean. **E**) Each symbol represents data from an individual donor [LF donors with 20-40 years of age (n = 4), LF donors with 55-65 years of age (n = 4), HF donors with 64-69 years of age (n = 4), and HF donors with 70-82 years of age (n = 8)], horizontal lines indicate group mean.


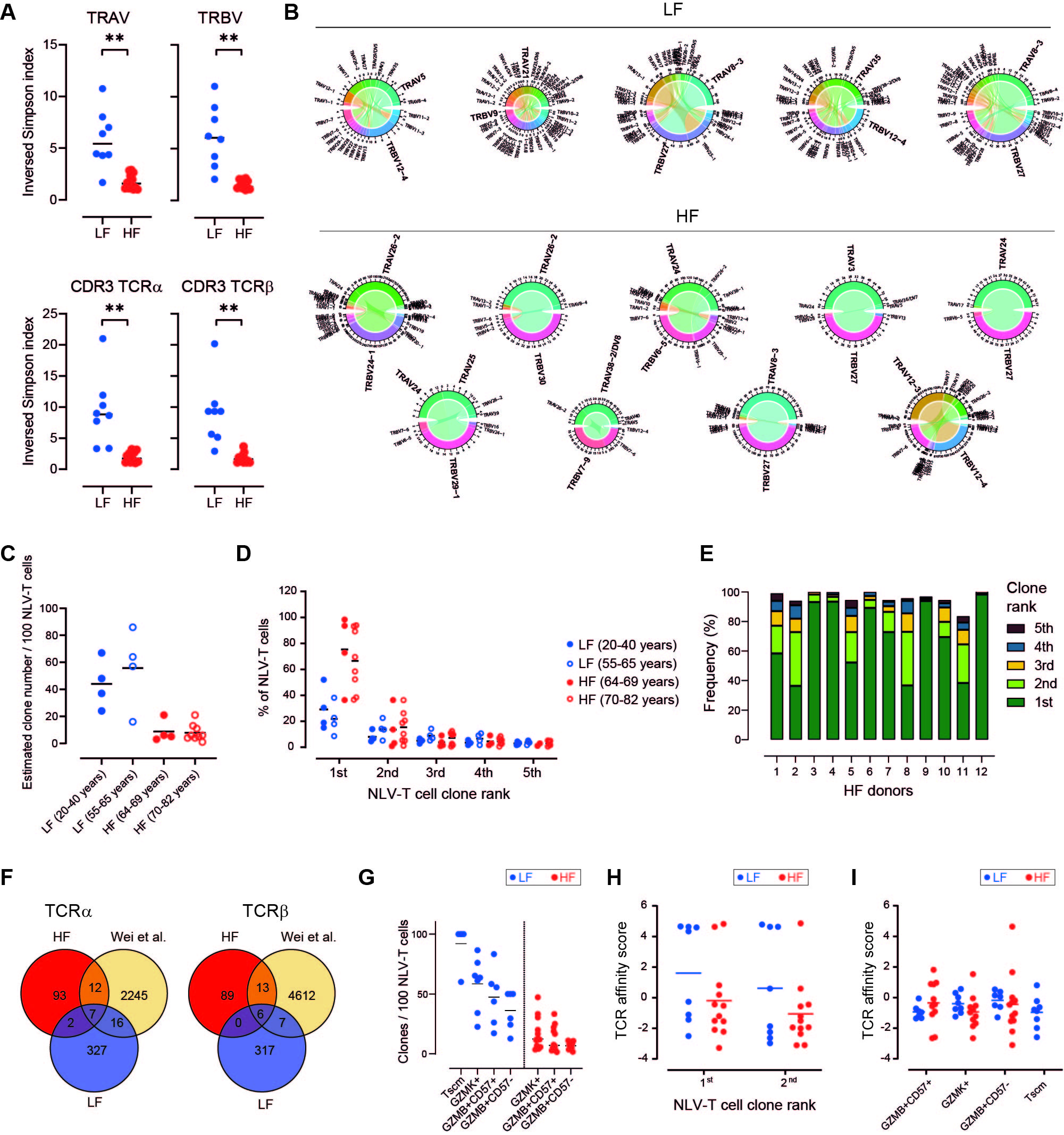


**Fig. S5. Detailed analysis of NLV-T cell clones, related to Fig. 3.**

**A**) Inverted Simpson indices for TRAV and TRBV genes (upper row) and CDR3 regions of TCRα and TCRβ genes (lower row). Unpaired t-test with Welch’s correction: ** p < 0.01. **B**) Circos plots depicting TRAV-TRBV gene pairing in NLV-T cells from the remaining 5 LF and 9 HF donors not depicted in Figure 3b. The top TRAV-TRBV pair in each donor is highlighted. **C**) NLV-T cell clonotype diversity expressed as the estimated number of unique clones per 100 cells in indicated age-subgroups of LF and HF donors. **D**) Frequency of top five clones within total NLV-T cell number from each donor in indicated age-subgroups of LF and HF donors. **E**) Frequency of top five NLV-T cell clones from each HF donor. **F**) Venn diagrams showing TCRα and TCRβ gene expression overlaps among NLV-T cells from LF and HF donors and publicly available dataset [57]. **G**) The estimated number of unique donor-specific clones per 100 NLV-T cells from LF and HF donors within each indicated Seurat cluster. **H**) TCR affinity scores of the top two expanded NLV-T cell clones from each donor were calculated using the pMTnet package. Two-way ANOVA (factors: Donor group and Clone rank) followed by Sidak’s multiple comparisons test, p> 0.05. **I**) Average affinity score of all NLV-T cell clones within each indicated cluster calculated using the pMTnet package Two-way ANOVA (factors: Donor group and Cluster) followed by Sidak’s multiple comparisons test, p> 0.05. **A**, **C**, **D**, **G - I**) Each symbol represents data from an individual donor (n= 8 and 12 for the LF and HF groups, respectively); horizontal lines indicate group mean.


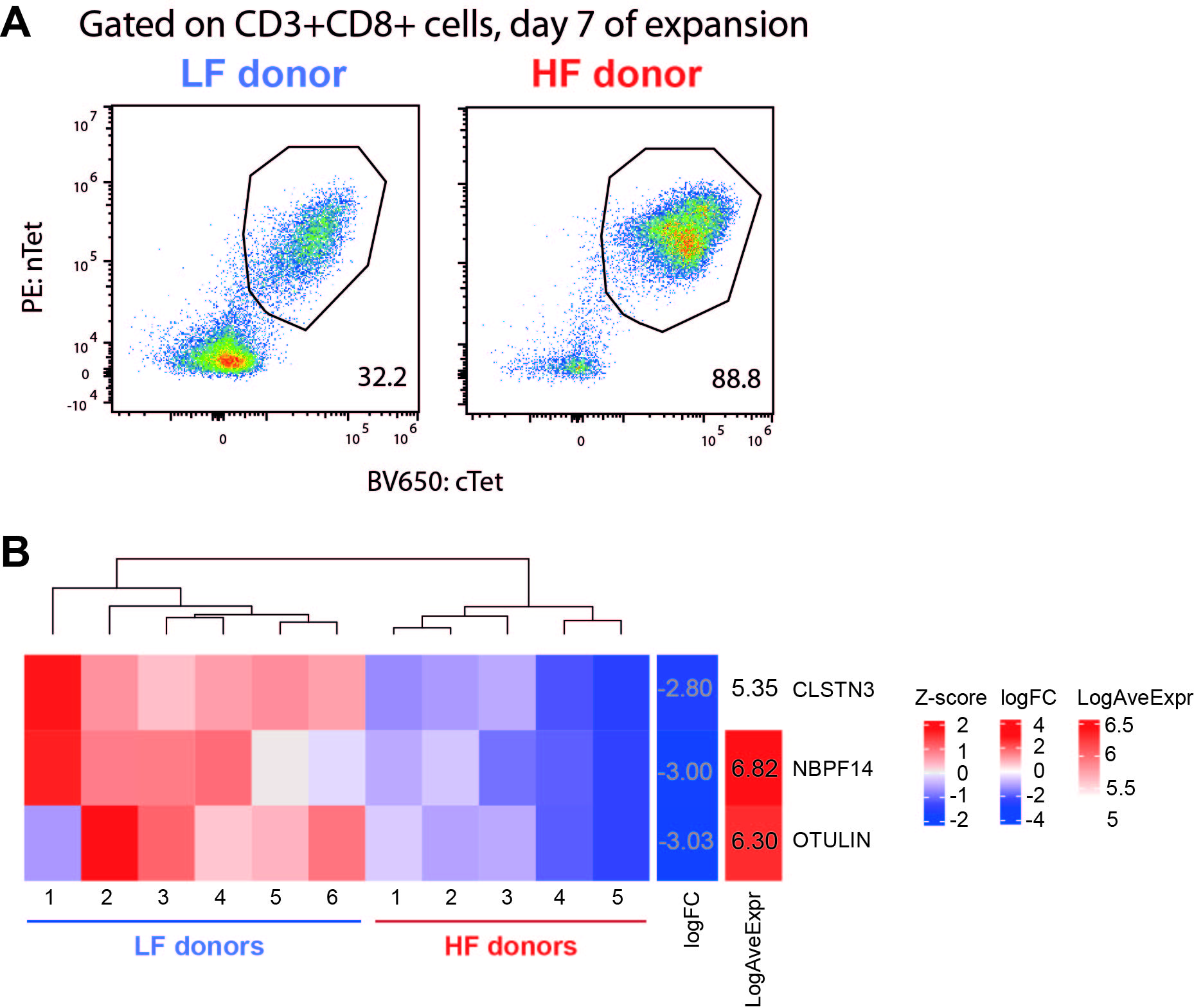


**Fig. S6. Additional information on *ex vivo* expanded NLV-T cells, related to Fig. 4.**

**A**) Spectral flow cytometry data showing frequency of T cells at day 7 of expansion stained with conventional (cTet) and null-tetramers (nTet) NLV (NLV-T cells) within CD3+CD8+ cells. Cells were labelled with Panel 1a antibodies from Table S2 and gated as depicted in Fig. S1a. Representative examples from one LF and one HF donor. **B**) A heatmap showing differentially expressed genes between NLV-T cells from LF and HF donors detected with bulk RNA sequencing (n= 8 and 12 for the LF and HF groups, respectively). Legend: LogFC - log_2_(fold change); LogAveExpr - log_10_(average gene expression).


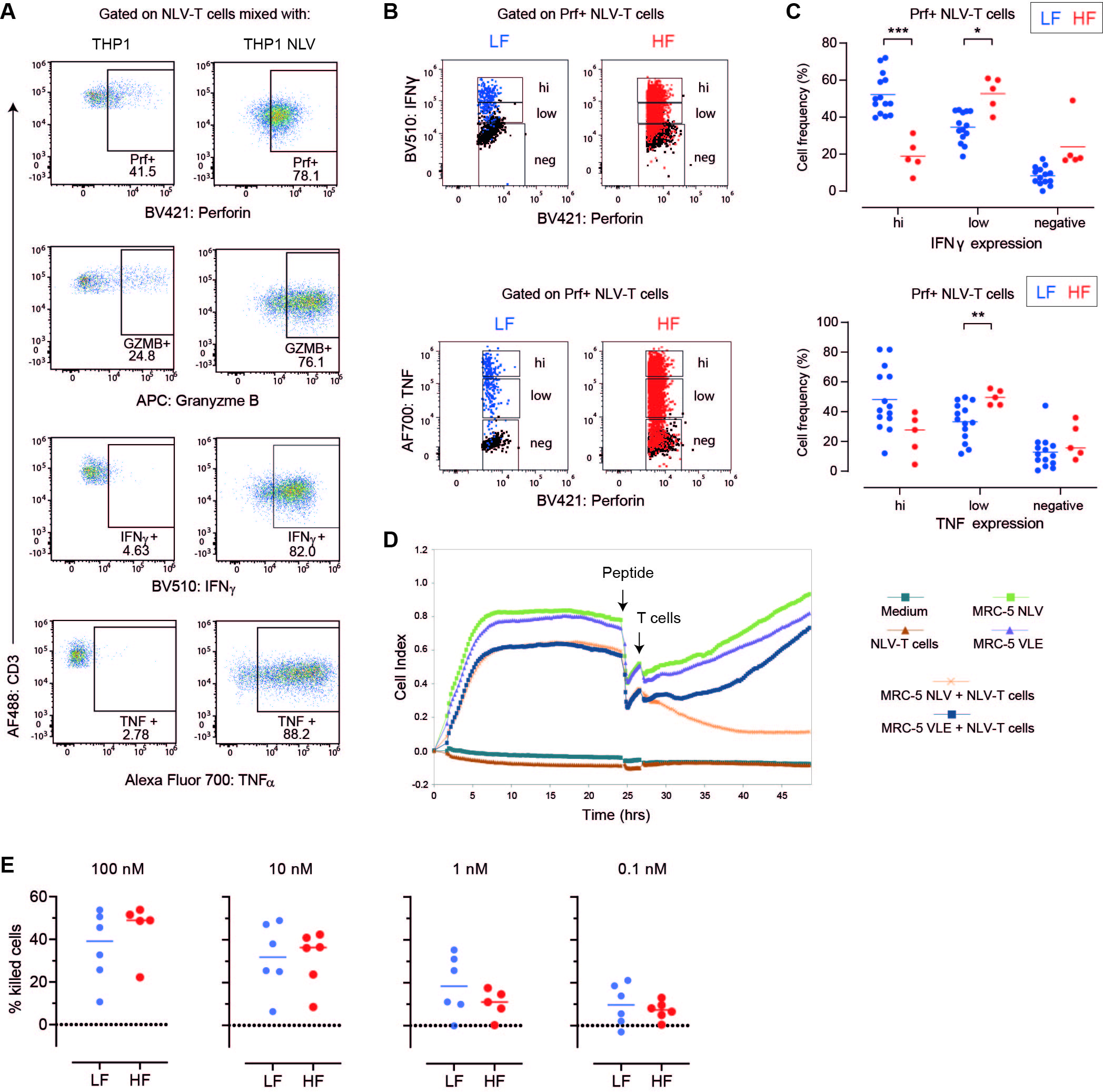


**Fig. S7. Additional information on NLV-T cell effector molecule production, degranulation, and killing of peptide-loaded targets, related to Fig. 6.**

**A**) Representative pseudocolor plots showing gating strategy for the analysis of perforin, granzyme B, interferon-gamma (IFNγ), and tumor necrosis factor (TNF) in the NLV-restimulated NLV-T cells according to the staining within the CD3+ CD8- population. **B**) Representative dot plots showing IFNγ and TNF staining on Perforin (Prf)+ NLV-T cells from an LF and an HF donor incubated with non-loaded (black dots) or NLV peptide-loaded (blue or red dots) THP-1 targets. NLV-T cell gating as shown in Fig. S6A. **C**) Distribution of Prf+ NLV-T cells from 14 LF and 5 HF donors according to the IFNγ and TNFα production. Two-way ANOVA (factors: Donor group and Cytokine expression level) followed by Sidak’s multiple comparisons test; * p<0.05, *** p<0.001. **D**) A representative example of the functional avidity assay using an impedance-based killing assay. MRC-5 cells were grown on 96-well plates that measured electric resistance (impedance) to determine cell density. Cell index values indicate MRC-5 cell attachment and growth for the first 24 hours when antigen-specific HCMV pp65 (495-503) HLA-A*0201 NLVPMVATV (NLV) peptide or antigen-non-specific HCMV IE-1 (316-324) HLA-A*0201 VLEETSVML (VLE) peptide were added at the concentration of 10 µM to load HLA-A*02 molecules (marked by the first arrow). After 2 hours of peptide loading, the *ex vivo* expanded NLV-T cells were added at the rate of one T cell to four MCR-5 targets (marked by the second arrow). The CD8 T cell-mediated killing of MCR-5 target cells was measured as a decrease in the cell index (impedance) during the next 18 hours of culture. Data are presented as average cell index values of four wells per condition. Legend: *Medium only* - wells containing only medium, without cells; *NLV-T cells only* - wells in which only T cells were added, without MRC-5 targets; *MRC-5 NLV* and *MRC-5 VLE* - wells containing MRC-5 fibroblasts and loaded with indicated peptides without NLV-T cells; *MRC-5 NLV + NLV-T cells* and *MRC-5 VLE + NLV-T cells* - wells containing MRC-5 fibroblasts loaded with indicated peptides mixed with NLV-T cells. **E**) Efficacy of NLV-T cells in eliminating MRC-5 fibroblasts loaded with indicated amounts of NLV-peptide (n= 6 and 5 for the LF and HF groups, respectively). Unpaired t-test with Welch`s correction, p > 0.05.


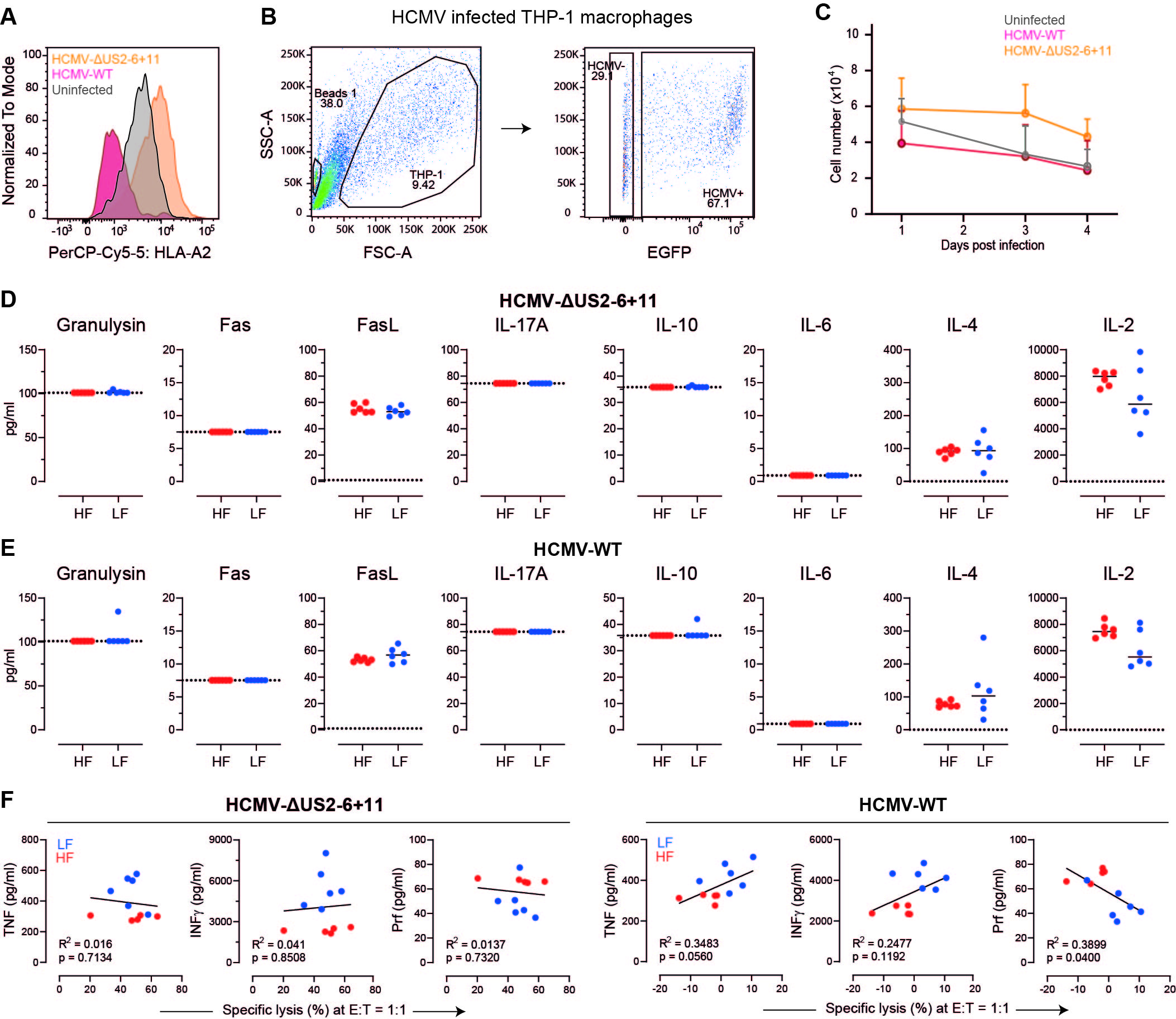


**Fig. S8. Additional information on NLV-T cell killing of HCMV-infected targets and impedance, related to Fig. 7.**

**A**) Histograms showing HLA-A2 antibody staining of uninfected (grey), HCMV-WT-infected (red), and HCMV-ΔUS2-6+11-infected (orange) THP-1 differentiated macrophages at 72 hours post infection. Representative of three independent experiments. **B**) Representative gating example of for detection of EGFP-expressing HCMV infected THP1-derived macrophages. Data are from THP-1 macrophages infected with HCMV-ΔUS2-6+11 four days post infection. **C**) Number of uninfected (grey), HCMV-ΔUS2-6+11-infected (red), and HCMV-WT-infected (orange) THP-1 cells at 1-4 days post-infection. Data are means displayed as means of three independent experiments ± standard deviation (SD). **D, E**) Concentrations of indicated effector molecules in the supernatants collected from the killing assay of (**D**) HCMV-WT- or (**E**) HCMV-ΔUS2-6+11-infected target cells with E:T 1:1 (n= 6 and 5 for the LF and HF groups, respectively). The dotted line on each graph represents the detection limit for a particular molecule. Unpaired t-test with Welch`s correction: p > 0.05. **F**) Correlations of NLV-T cell specific lysis of HCMV-infected targets with concentrations of indicated mediators in supernatants. Dots - data from individual donors, line - simple linear regression.) Each symbol represents data from an individual donor; horizontal lines indicate group mean.

**Table S1. Overview of the samples included in the present study.**

| Age | Male | Female | Total sample number |
| --- | --- | --- | --- |
| 20-59 years | 18 (58.1%) | 13 (41.9%) | 31 |
| 60-88 years | 44 (51.7%) | 41 (48.3%) | 85 |

**Table S2. List of staining reagents.** Legend: AF – Alexa Fluor; APC - Allophycocyanin; BB – Brilliant Blue; BUV – Brilliant Ultra Violet; BV - Brilliant Violet; Cy – Cyanine; N/A – not applicable; PE – Phycoerythrin; PerCP - Peridinin-Chlorophyll-Protein.

| **Target** | **Conjugate** | **Clone or Totalseq code** | **Source** | **Identifier** | **Dilution** |
| --- | --- | --- | --- | --- | --- |
| ***Panel 1a – characterization of NLV-T cells in PBMC samples*** | | | | | |
| CD45RA | BUV395 | HI100 | BD | Cat# 740298  RRID:AB_2740037 | 1:200 |
| CD16 | BUV496 | 3G8 | BD | Cat# 612944  RRID:AB_2870224 | 1:100 |
| CD4 | BUV563 | RPA-T4 | BD | Cat# 741353  RRID:AB_2870854 | 1:200 |
| CD56 | BUV661 | B159 | BD | Cat# 741618  RRID:AB_2871025 | 1:100 |
| Lag-3 | BV421 | 11C3C65 | BioLegend | Cat# 369314  RRID:AB_2629797 | 1:100 |
| CD57 | Pacific Blue | HNK-1 | BioLegend | Cat# 359608  RRID:AB_2562459 | 1:100 |
| CD45RO | BV570 | UCHL1 | BioLegend | Cat# 304226  RRID:AB_2563818 | 1:100 |
| CD19 | BV510 | HIB19 | BioLegend | Cat# 302242  RRID:AB_2561668 | 1:50 |
| Tim-3 | BV605 | F38-2E2 | BioLegend | Cat# 345018  RRID:AB_2563859 | 1:200 |
| CD27 | BV785 | O323 | BioLegend | Cat# 302832  RRID:AB_2562674 | 1:100 |
| CD3 | AF488 | UCHT1 | BioLegend | Cat# 300415  RRID:AB_389310 | 1:100 |
| CD14 | BB700 | MoP9 | BD | Cat# 566465  RRID:AB_2739737 | 1:100 |
| TIGIT | PE-eFluor610 | MBSA43 | Thermo Fisher Scientific | Cat# 61-9500-42  RRID:AB_2723715 | 1:100 |
| CD28 | PE-Cy5 | CD28.2 | BioLegend | Cat# 302910  RRID:AB_314312 | 1:100 |
| CTLA-4 | PE-Cy7 | L3D10 | BioLegend | Cat# 349914  RRID:AB_2563098 | 1:100 |
| CD8 | APC | SK1 | BioLegend | Cat# 344722  RRID:AB_2075388 | 1:100 |
| CCR7 | APC-R700 | 2-L1-A | BD | Cat# 566766  RRID:AB_2869856 | 1:100 |
| PD-1 | APC-Cy7 | EH12.2H7 | BioLegend | Cat# 329922  RRID:AB_10933429 | 1:100 |
| Empty loadable tetramer  HLA-A*0201 (cTet) | BV650 | N/A | Tetramer Shop | Cat# HA02-070 | 1:10 |
| iTAg Tetramer - HLA-A*02:01 CMV pp65 498-503 (NLVPMVATV) (nTet) | PE | N/A | MBL International Corporation | Cat# TB-0010-1 | 1:10 |
| Zombi NIR^TM^ dye | | N/A | BioLegend | Cat# 423105 | 1:400 |
| ***Panel 1b – characterization of NLV-T cells following* ex vivo *expansion*** | | | | | |
| CD45RA | BUV395 | HI100 | BD | Cat# 740298  RRID:AB_2740037 | 1:200 |
| CD16 | BUV496 | 3G8 | BD | Cat# 612944  RRID:AB_2870224 | 1:100 |
| CD4 | BUV563 | RPA-T4 | BD | Cat# 741353  RRID:AB_2870854 | 1:200 |
| CD56 | BUV661 | B159 | BD | Cat# 741618  RRID:AB_2871025 | 1:100 |
| Lag-3 | BV421 | 11C3C65 | BioLegend | Cat# 369314  RRID:AB_2629797 | 1:100 |
| CD57 | Pacific Blue | HNK-1 | BioLegend | Cat# 359608  RRID:AB_2562459 | 1:100 |
| CD45RO | BV570 | UCHL1 | BioLegend | Cat# 304226  RRID:AB_2563818 | 1:100 |
| CD19 | BV510 | HIB19 | BioLegend | Cat# 302242  RRID:AB_2561668 | 1:50 |
| Tim-3 | BV605 | F38-2E2 | BioLegend | Cat# 345018  RRID:AB_2563859 | 1:200 |
| CD27 | BV785 | O323 | BioLegend | Cat# 302832  RRID:AB_2562674 | 1:100 |
| CD3 | AF488 | UCHT1 | BioLegend | Cat# 300415  RRID:AB_389310 | 1:100 |
| CD14 | BB700 | MoP9 | BD | Cat# 566465  RRID:AB_2739737 | 1:100 |
| TIGIT | PE-eFluor610 | MBSA43 | Thermo Fisher Scientific | Cat# 61-9500-42  RRID:AB_2723715 | 1:100 |
| CD28 | PE-Cy5 | CD28.2 | BioLegend | Cat# 302910  RRID:AB_314312 | 1:100 |
| CTLA-4 | PE-Cy7 | L3D10 | BioLegend | Cat# 349914  RRID:AB_2563098 | 1:100 |
| CD8 | APC | SK1 | BioLegend | Cat# 344722  RRID:AB_2075388 | 1:100 |
| CCR7 | APC-R700 | 2-L1-A | BD | Cat# 566766  RRID:AB_2869856 | 1:100 |
| PD-1 | APC-Cy7 | EH12.2H7 | BioLegend | Cat# 329922  RRID:AB_10933429 | 1:100 |
| HLA-A*02:01 CMV pp65 498-503 (NLVPMVATV) (cTet) | PE | N/A | NIH Tetramer Core Facility of Emory University, (Atlanta, GA) | N/A | 1:800 |
| Zombi NIR^TM^ dye | | N/A | BioLegend | Cat# 423105 | 1:400 |
| ***Panel 2 – NLV-T cell single cell RNA sequencing*** | | | | | |
| CD3 | AF488 | UCHT1 | BioLegend | Cat# 300415  RRID:AB_389310 | 1:100 |
| CD8 | APC-Cy7 | SK1 | BioLegend | Cat# 344714  RRID:AB_2044006 | 1:100 |
| CD14 | BB700 | MoP9 | BD | Cat# 566465  RRID:AB_2739737 | 1:100 |
| CD19 | BV510 | HIB19 | BioLegend | Cat# 302242  RRID:AB_2561668 | 1:50 |
| CD56 | PE-Cy7 | NCAM16.2 | BD | Cat# 335826  RRID:AB_2857328 | 1:100 |
| CD16 | BV421 | 3G8 | BioLegend | Cat# 302038  RRID:AB_2561578 | 1:100 |
| TCR γδ | APC | B1 | BioLegend | Cat# 331212  RRID:AB_1089214 | 1:100 |
| HLA-A*02:01 CMV pp65 498-503 (NLVPMVATV) (cTet) | PE | N/A | NIH Tetramer Core Facility of Emory University, (Atlanta, GA) | N/A | 1:800 |
| CD57 | N/A | TotalSeq™  C0168 | Biolegend | Cat# 393321 RRID:AB_2801030 | 1:100 |
| CD45RO | N/A | TotalSeq™ C0087 | Biolegend | Cat# 304259 RRID:AB_2800766 | 1:100 |
| CD27 | N/A | TotalSeq™ C0154 | Biolegend | Cat# 302853 RRID:AB_2800747 | 1:200 |
| CD45RA | N/A | TotalSeq™ C0063 | Biolegend | Cat# 304163 RRID:AB_2800764 | 1:200 |
| anti-human Hashtag 1 | N/A | TotalSeq™ C0251 | Biolegend | Cat# 394661 RRID:AB_2801031 | 1:100 |
| anti-human Hashtag 2 | N/A | TotalSeq™ C0252 | Biolegend | Cat# 394663 RRID:AB_2801032 | 1:100 |
| anti-human Hashtag 4 | N/A | TotalSeq™ C0254 | Biolegend | Cat# 394667 RRID:AB_2801034 | 1:100 |
| anti-human Hashtag 5 | N/A | TotalSeq™ C0255 | Biolegend | Cat# 394669 RRID:AB_2801035 | 1:100 |
| anti-human Hashtag 6 | N/A | TotalSeq™ C0256 | Biolegend | Cat# 394671 RRID:AB_2820042 | 1:100 |
| anti-human Hashtag 7 | N/A | TotalSeq™ C0257 | Biolegend | Cat# 394673 RRID:AB_2820043 | 1:100 |
| anti-human Hashtag 8 | N/A | TotalSeq™ C0258 | Biolegend | Cat# 394675 RRID:AB_2820044 | 1:100 |
| anti-human Hashtag 9 | N/A | TotalSeq™ C0259 | Biolegend | Cat# 394677 RRID:AB_2820045 | 1:100 |
| ***Panel 3 – NLV-T cell effector molecules*** | | | | | |
| Perforin | BV421 | δG9 | BD | Cat# 563393  RRID:AB_2738178 | 1:50 |
| TNFα | AF700 | MAb11 | BioLegend | Cat# 502928  RRID:AB_2561315 | 1:50 |
| IFNγ | BV510 | B27 | BioLegend | Cat# 506540  RRID:AB_2801100 | 1:100 |
| Granzyme B | APC | GB11 | Invitrogen | Cat# GRB05  RRID:AB_2536539 | 1:50 |
| CD3 | AF488 | UCHT1 | BioLegend | Cat# 300415  RRID:AB_389310 | 1:100 |
| CD8 | BUV563 | RPA-T8 | BD | Cat# 612914  RRID:AB_2870199 | 1:100 |
| HLA-A*02:01 CMV pp65 498-503 (NLVPMVATV) (cTet) | PE | N/A | NIH Tetramer Core Facility of Emory University, (Atlanta, GA) | N/A | 1:800 |
| LIVE/DEAD Fixable Violet Dead Cell Stain Kit | | N/A | Thermo Fisher Scientific | Cat# L34964 | 1:400 |
| ***Panel 4 – NLV T cell degranulation*** | | | | | |
| CD107a (LAMP-1) | PE | H4A3 | BioLegend | Cat# 328608 RRID:AB_1186040 | 1:100 |
| CD3 | AF488 | UCHT1 | BioLegend | Cat# 300415  RRID:AB_389310 | 1:100 |
| CD8 | BUV563 | RPA-T8 | BD | Cat# 612914  RRID:AB_2870199 | 1:100 |
| HLA-A*02:01 CMV pp65 498-503 (NLVPMVATV) (cTet) | APC | N/A | NIH Tetramer Core Facility of Emory University, (Atlanta, GA) | N/A | 1:400 |
| ***Additional staining markers*** | | | | | |
| CellTracker Green CMFDA | | N/A | Thermo Fisher Scientific | Cat# C2925 | 1:5000 |
| CellTracker Deep Red | | N/A | Thermo Fisher Scientific | Cat# C34565 | 1:10000 |
| HLA-A2 | PerCP-Cy 5.5 | BB7.2 | Biolegend | Cat# 343316 RRID:AB_2561573 | 1:100 |

**Supplementary reference**

Lu, J., Ahmad, R., Nguyen, T., Cifello, J., Hemani, H., Li, J., Chen, J., Li, S., Wang, J., Achour, A., Chen, J., Colie, M., Lustig, A., Dunn, C., Zukley, L., Chia, C.W., Burd, I., Zhu, J., Ferrucci, L., and Weng, N.-P. 2022. Heterogeneity and transcriptome changes of human CD8+ T cells across nine decades of life. *Nat. Commun.* 13, 5128. doi:10.1038/s41467-022-32869-x.
